# Understanding the value of meningococcal vaccination for adolescents and young adults in the United States: insights from a steady-state modelling approach

**DOI:** 10.1186/s12889-025-21953-8

**Published:** 2025-05-20

**Authors:** E. Langevin, C. Robertson, K. Galarza, A. Dogu, O. Cristeau, E. Clay, J. Wu, T. Shin

**Affiliations:** 1 Sanofi Vaccines, Lyon, France; 2 Sanofi Vaccines, Swiftwater, PA USA; 3 Sanofi Vaccines, Dubai, United Arab Emirates; 4 Clever-Access, Paris, France; 5 Department of Mathematics and Statistics, York University, Toronto, Canada; 6 Sanofi Vaccines, Toronto, Canada

## Abstract

**Background:**

A two-dose series of quadrivalent meningococcal conjugate vaccine (MenACWY) is recommended for the prevention of invasive meningococcal disease (IMD) in adolescents in the United States. In June 2024, the Advisory Committee on Immunization Practices discussed plans to review the adolescent meningococcal vaccination schedule. Various options are under consideration, including removing the first dose of MenACWY at age 11–12 years.

**Objectives:**

We evaluated the public health impact and cost-effectiveness of administering one or two doses of MenACWY compared to a scenario with no vaccination.

**Methods:**

We constructed an incidence-based population model to compare costs and quality-adjusted life years (QALYs) associated with different vaccination schedules in a cohort of 11–25 year-olds, from a societal perspective, over a lifetime analytic horizon for outcomes related to death and disabilities. The main analyses compared various scenarios of MenACWY (Q) and MenB schedules to no vaccination. Further scenarios examined the impact of alternative assumptions applied to the first and/or second dose of MenACWY.

**Results:**

Compared to no vaccination, 2 doses of MenACWY and 2 doses of MenB vaccine was projected to reduce IMD cases by 277 per year, resulting in an incremental cost-effectiveness ratio (ICER) of $625,322/QALY. Administering 2 doses of MenACWY was projected to reduce the annual number of IMD cases by 275 at an ICER of $438,948/QALY, which increased to 631 at an ICER of $190,030/QALY when herd immunity was considered. Alternatively, if only 1 dose of MenACWY was administered, the reduction in cases would be 253 if administered at 11–12 years old and 125 if given at 16 years, with ICERs of $252,249 per QALY and $352,169/QALY, respectively. Assuming a 25% increase in vaccination coverage rate, one MenACWY dose at 16 years resulted in 156 cases avoided.

**Conclusions:**

The two doses of MenACWY that are currently recommended play a crucial role in reducing the burden of IMD and the first dose contributes significantly (≥ 90%) to this reduction. It is essential to take this finding into account when considering any updates to the adolescent meningococcal vaccination schedule in the United States.

**Supplementary Information:**

The online version contains supplementary material available at 10.1186/s12889-025-21953-8.

## Background

Invasive meningococcal disease (IMD) is a serious bacterial infection caused by Neisseria meningitidis, manifesting as meningitis or septicemia [1]. Worldwide, IMD is primarily caused by serogroups A, B, C, W, and Y, with B, C, and Y most common in the United States (US) [2]. Approximately 10% of people are asymptomatic carriers, with adolescents having the highest carriage rates [2, 3].

IMD carries a high mortality risk, with 5–10% of patients dying within 24–48 h of symptom onset, and about a third of deaths occurring post-discharge [4, 5]. Among survivors, 10–20% may suffer severe long-term sequelae, including neurological disabilities, renal damage, amputations, and hearing loss among others [5, 6].

In 2023, 483 IMD cases were reported to the Centers for Disease Control and Prevention (CDC), the highest since 2013 [7]. In March 2024, the CDC issued a health advisory due to rising Y serogroup IMD, including antibiotic-resistant strains [7, 8].

The highest IMD incidence in the US is in infants, with a second peak in adolescents and young adults (AYA) [7]. AYAs have increased risks of death and long-term sequelae [9], the highest oropharyngeal carriage rates, and are primary transmission sources [3]. The case-fatality rate among AYAs aged 16–23 years was 7.5–11.8% in 2019–2022 [10–13].

Five meningococcal vaccines are available in the US: two MenACWY vaccines (Menveo^®^ and MenQuadfi^®^), two MenB vaccines (Bexsero^®^ and Trumenba^®^), and a pentavalent MenABCWY vaccine (PenbrayaTM) [14]. Another pentavalent is planned to be marketed in the first quarter of 2025.

Clinical trials showed high seroprotection rates after one MenACWY dose (93.5% for serogroup A to 99.1% for W) [15, 16], with booster doses maintaining protection above 90% for all serogroups [17]. In 2005, ACIP recommended MenACWY vaccine for adolescents aged 11–18 years, with the first dose at 11–12 years. In 2010, ACIP added a booster dose at 16 years. In 2022, 88.6% of adolescents received ≥ 1 MenACWY dose, and 60.8% received ≥ 2 doses [18].

MenB vaccination, a two- or three-dose series, was recommended in 2015 under shared clinical decision-making for AYAs aged 16–23 years [16]. In 2022, coverage was 29.4% for ≥ 1 dose and 11.9% for ≥ 2 doses [18].

In April 2024, the CDC published ACIP’s recommendations for MenABCWY use in individuals aged ≥ 10 years [17]. The vaccine is indicated for healthy persons aged 16–23 years when shared clinical decision-making favors MenB and for those at increased IMD risk [19].

ACIP is discussing the adolescent meningococcal vaccination schedule, considering alternatives with fewer MenACWY doses [20, 21]. This study aims to inform ACIP’s decision-making by evaluating the public health impact and cost-effectiveness of different vaccination programs.

## Methods

### Model overview

**Fig. 1:**
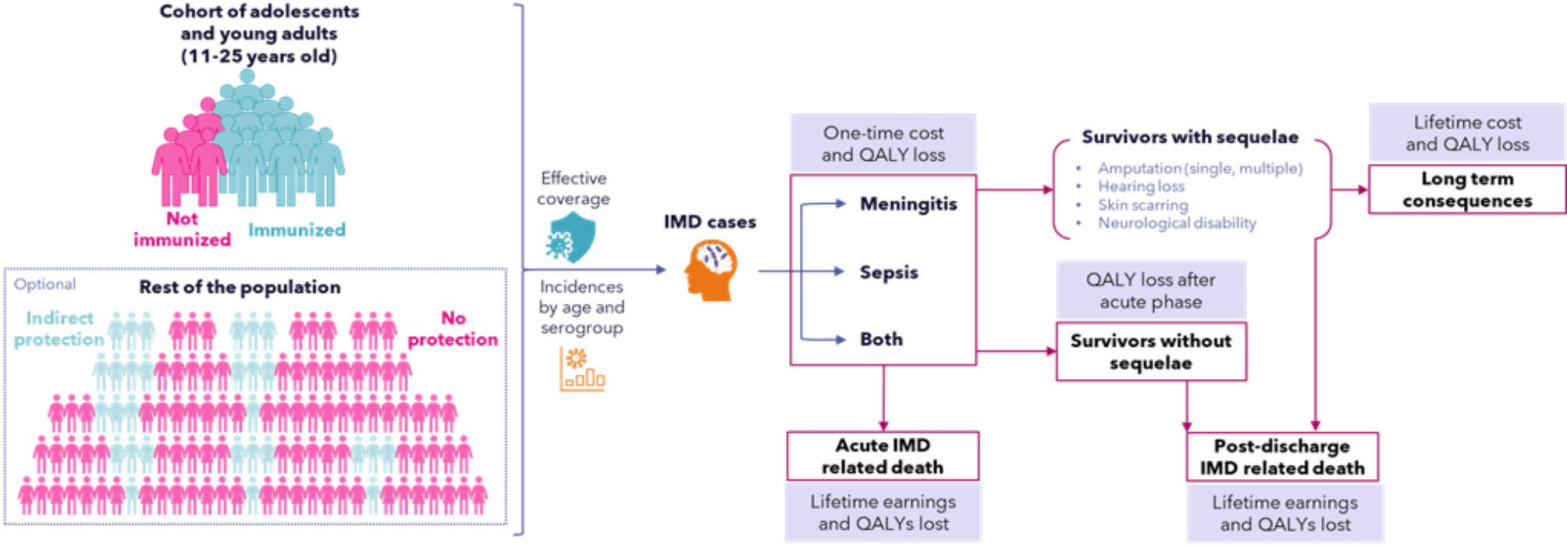
Model schematic. Abbreviations: IMD, invasive meningococcal disease; QALY, quality-adjusted life-year

Public health impact and cost-effectiveness of different meningococcal vaccination schedules were analyzed from the societal perspective i.e., including both direct and indirect costs. An incidence-based static population cost-effectiveness model was developed focusing on *N. meningitidis* serogroups A, B, C, W, and Y. The model simulated the steady state of two vaccination schedules in a cohort of AYA aged 11–25 years (or entire population for the scenario that considered herd immunity), and compared their impact on the epidemiology, costs, and humanistic burden of IMD in the US. The model assumed a constant population and constant transmission intensity to estimate the number of IMD cases, by age and serogroup, occurring in a routine year of when the different vaccination schedules are in place. Long-term consequences of IMD were considered and discounted over a lifetime horizon. A schematic overview of the model is presented in Fig. 1, and details on methods and data are available in the Supplementary Methods. All costs are provided in 2023 USD.

### Vaccination schedules

The model compared each vaccination schedule of interest to a “No vaccination” strategy. The four vaccination schedules assessed in the model are listed in Table 1 and include the current US immunization schedule in which MenACWY is given at 11–12 and 16 years of age and MenB at 16 and 16.5 years of age [22] (Q-QB-B schedule), the Q-Q schedule in which the Men B vaccination is omitted and MenACWY is administered as per current recommended schedule, and two schedules considering each dose of MenACWY given separately (Q-N for a single dose at 11 years and N-Q for a single dose at 16 years). These schedules allowed us to evaluate the value of the current adolescent meningococcal vaccination schedule as a whole and the value of each dose when given independently.

**Table 1:** Vaccination schedules considered in the study

Vaccine schedule	First dose (Age 11–12)	Second dose (Age 16)
No vaccine	None	None
Q-QB-B	MenACWY	MenACWY + 2 doses of MenB (at 16 and 16.5 years of age)
Q-Q	MenACWY	MenACWY
Q-N	MenACWY	None
N-Q	None	MenACWY
Q-P-B (scenario)	MenACWY	MenABCWY at 16 years + Men B at 16.5 years

Model inputs are summarized in Table 2 and more information is available in the Supplementary Methods and in Table S1.

**Table 2:** Key inputs of the model

Parameter	Parameter characteristics	Value	Source
Life expectancy (years)	Women	79.3	CDC National Vital Statistics Reports [23]
	Men	73.5	
Proportion of women		50.4%	US Census Bureau [24]
Population size by age group	11–17 years	29,878,732	US Census Bureau [25]
	18–25 years	35,674,439	
IMD rates (per 100,000 individuals)	11–17 years	A: 0.0 B: 0.974 C: 0.3356 W: 0.0 Y: 0.2707	US surveillance data [26] and Shepard et al. [27]
	18–25 years	A: 0.0 B: 0.2256 C: 0.3652 W: 0.0079 Y: 0.1809	
Distribution of clinical presentation of IMD	Meningitis	50.6%	Davis et al. [28]
	Septicemia	33.4%	
	Other meningococcal infection	16.0%	
Probability of occurrence of permanent sequelae	Skin scarring	7.6%	Ortega-Sanchez [26]
	Single amputation	1.9%	
	Multiple amputation	1.2%	
	Hearing loss	8.8%	
	Significant long term neurologic disability	2.1%	
Case fatality rate (CFR) in the acute phase of serogroup B disease	11–17 years	8.7%	NNDSS [7]
	18–25 years	9.2%	
Case fatality rate in the acute phase of serogroup CWY disease	11–17 years	12.3%	
	18–25 years	18.3%	
Excess mortality in survivors (IRR of post-discharge to acute CFR applied to CFRs presented above)	IMD without sequelae	0.422	Shen et al. [4] CFR: 8.3% Morality rate post-discharge: without sequelae: 3.5%; with sequelae: 8.4%
	IMD with sequelae	1.012	
Vaccination coverage rates (MenACWY)	11 years	88.6%	2022 National Immunization Survey [18]
	16 years	60.8%	
Vaccination coverage rates (MenB)	16 years	29.4%	
	16.5 years	11.9%	
Vaccination coverage (scenario Q-P-B)	11 years (MenACWY)	88.6%	2022 National Immunization Survey [18]
	16 years (MenABCWY)	60.8%	
	16.5 years (MenB)	30.4%	Assumption 50% of VCR at 16 years
Vaccine efficacy (first dose / two-dose course)	MenB	0.0% / 85.0%^a^	Ortega-Sanchez [26]
	MenACWY	97.0% / 97.0%^a^	Chang et al. [16] and Dhingra et al. [15]
Annual waning rate	MenACWY	3.0% (linear)	Assumption based on clinical data [17]
	MenB	33.3% (exponential)	Assumption
Indirect protection		35.0%	Ramsay et al. [29]
Vaccination costs (USD)	MenACWY	169.78	CDC Vaccine Price List [30] and assumptions from Ortega-Sanchez [26]
	MenB	219.11	
	MenABCWY	244.58	
Direct costs in the acute phase (hospitalization) (USD)	Meningitis	81,741.24	Davis et al. [28]
	Septicemia	115,841.55	
	Other meningococcal infection	101,269.73	
Public health response costs (USD)		13,604.56	Ortega-Sanchez [31]
Direct costs of sequelae^b^ (USD)	Skin scarring	7,467.59	Ortega-Sanchez [26]
	Single amputation	204,360.57	
	Multiple amputation	245,234.49	
	Hearing loss	89,946.53	
	Significant long term neurologic disability– 0–17years	2,921,074.90	
	Significant long term neurologic disability– ≥18 years	2,716,194.00	
Value of work time lost by caregivers in the acute phase (USD)		4,298.24	Ortega-Sanchez [26]
Market productivity (earnings) (USD)	11–17 years	5,556.44	Grosse et al. [32]
	18–25 years	17,471.37	
Non-market productivity (household services), applied to IMD death only (USD)	11–17 years	5,619.62	Grosse et al. [32]
	18–25 years	15,090.45	
Productivity loss	Skin scarring	0.0%	Ortega-Sanchez et al. [33]
	Single amputation	20.0%	
	Multiple amputation	30.0%	
	Hearing loss	33.0%	
	Significant long term neurologic disability	100.0%	
Baseline utility	Aged < 25	0.92	Jiang et al. [34]
	Overall population (scenario with herd effect)	0.851	
Acute IMD-related disutility	Meningitis	0.40	Lecoq et al. [35]
	Septicemia	0.51	
	Other meningococcal infection	0.40	
Duration of acute disutility		1 year (365.25 days)	Ortega-Sanchez [31]
Disutility for IMD cases without sequalae after acute phase	Disutility	0.03	Koomen et al. [36] and Schmand et al. [37]
	Duration	9 years	
Disutility with IMD sequalae	Skin scarring	0.05	Ortega-Sanchez [26]
	Single amputation	0.28	
	Multiple amputation	0.36	
	Hearing loss	0.26	
	Significant long term neurologic disability	0.86	

^a^ The same efficacy against the respective serogroups was assumed for MenABCWY in a scenario analysis

^b^ Lifetime costs

Abbreviations: IMD, invasive meningococcal disease; IRR: incidence rates ratio

Coverage rates currently observed in the US were retrieved from the 2022 National Immunization Survey published by the CDC and were considered in all scenarios [18].

### Vaccine efficacy

The efficacy of MenACWY was based on 30-day hSBA seroprotection data in adolescents (10–17 years) from phase 2 and phase 3 clinical trials for MenACWY-TT (MenQuadfi^®^, Sanofi) [15, 16]. The model assumed vaccine effectiveness of 97% for all serogroups. Vaccine efficacy was assumed to wane linearly by 3% annually based on data from a phase 3b open-label trial where 82.5% of participants were still seroprotected 3–6 years after the vaccination [17]. Further details are available in the Supplementary Methods.

For MenB, the model used similar assumptions as the CDC for vaccine efficacy [26]. The first dose of MenB was assumed not to provide any residual protection, while the efficacy of the full course of ≥ 2 doses was assumed to be 85% [26]. Exponential waning was assumed with an average duration of protection of 3 years.

### Sensitivity and scenario analyses

Scenario analyses were conducted to test additional vaccination schedules, deterministic sensitivity analysis (DSA) to identify the key drivers of model results, and probabilistic sensitivity analysis (PSA) to evaluate the impact of assumptions used in the model and uncertainty surrounding model inputs. PSA was run for 10,000 simulations. DSA and PSA inputs are available in Table S1.

Three scenarios were explored as part of these analyses. The first scenario included herd immunity, i.e., the indirect protection that could be expected from the current vaccination program with MenACWY. A 35% reduction in the number of IMD cases due to serogroups A, C,W, and Y in unprotected individuals was included, based on Ramsay et al. [29], where a decline of 35% of the IMD incidence in > 25 year-olds (not targeted by the vaccination) was observed in the UK following the introduction of a meningococcal immunization program. In order to assess the overall benefit of the vaccination, this scenario was conducted in the total US population. Baseline utility was adjusted to reflect the quality of life of the general population, as estimated by Jiang et al. [34]. Utility decrements for IMD sequelae were also adjusted to the overall US population.

The following two scenarios, both compared to no vaccination, aimed to inform decision makers on potential schedules that could be considered by the ACIP. In one scenario, the first dose of MenACWY was omitted, but the coverage rate of the dose administered at 16 years of age was increased by 25%, resulting in a vaccination coverage rate of 76%. In the second scenario, the Q-P-B schedule was analyzed in which the second dose of MenACWY and the first dose of MenB were replaced by a single dose of MenABCWY. This scenario assumed that MenABCWY would have the same coverage rate as currently exists for the quadrivalent vaccine, increasing the protection against the B serogroup compared to the current situation (a theoretical coverage rate of 60.8% vs. the current rate of 29.4% for the first dose). It was assumed that 50% of individuals receiving the first dose of MenABCWY would receive a second dose of MenB, approximating currently observed data for MenB alone [18]. The vaccination coverage rates (VCRs) and vaccine prices used in these scenarios are presented in Table 2. In line with the previous analyses presented to the ACIP, MenABCWY was assumed to have the same efficacy against the respective serogroups as the quadrivalent MenACWY and monovalent MenB vaccines [26, 31]. The publicly available price of Penbraya™ was considered in this analysis [30].

## Results

**Fig. 2:**
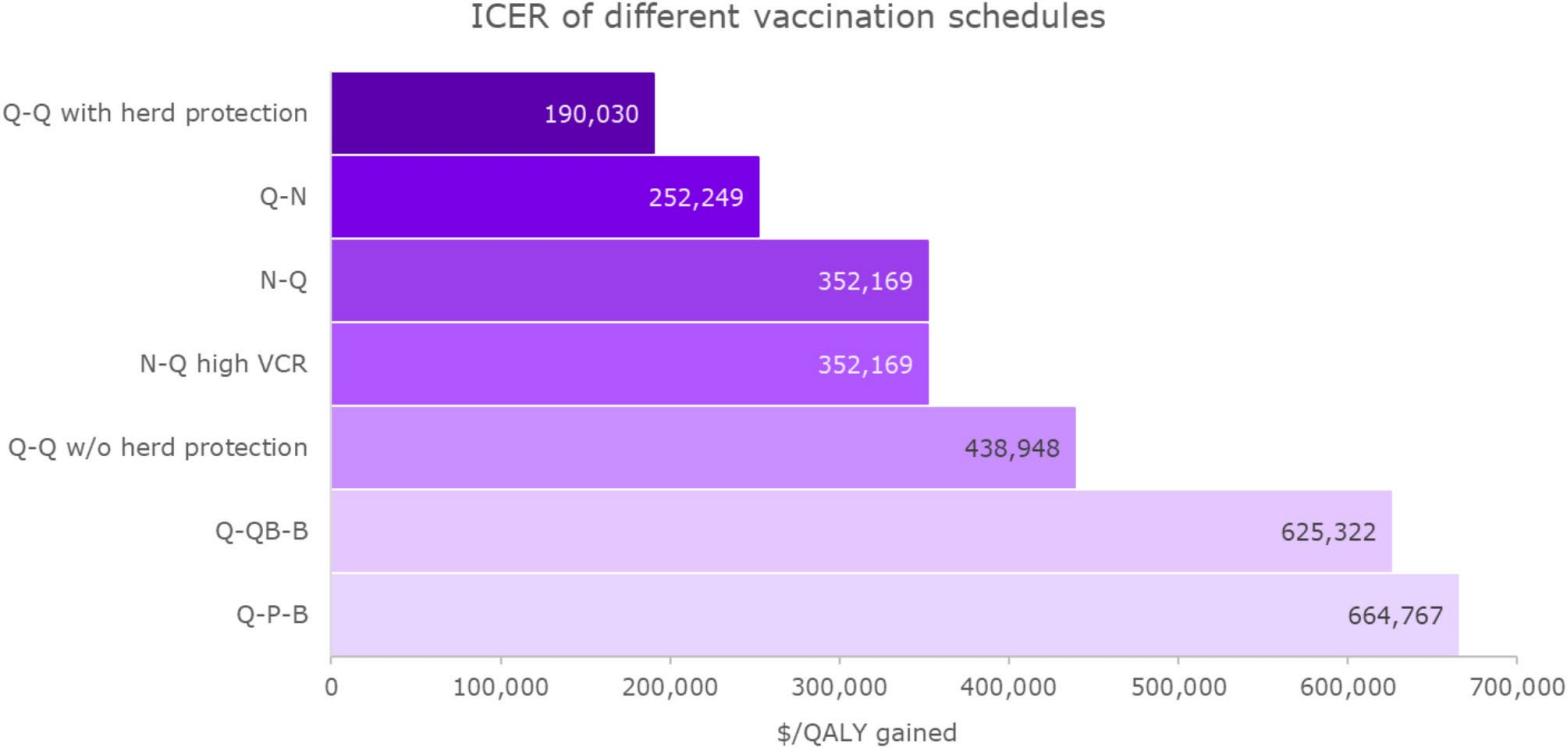
Incremental cost-effectiveness ratios of different vaccination schedules vs. no vaccination. Abbreviations: ICER, incremental cost-effectiveness ratio; QALY, quality-adjusted life-year; SoC, standard of care

The results of the base case analysis are presented in Table 3. Figure 2 summarizes the incremental costs effectiveness ratios (ICERs) vs. no vaccination for the different vaccination schedules tested.

**Table 3:** Base case results

		No vaccination	Incremental vs. No vaccination			
			Q-QB-B	Q-Q	Q-*N*	*N*-Q
Number of IMD cases	Total	488	-277	-275	-253	-125
	Serogroup A	0	0	0	0	0
	Serogroup B	110	-2	0	0	0
	Serogroup C	231	-166	-166	-152	-79
	Serogroup W	3	-2	-2	-2	-1
	Serogroup Y	145	-107	-107	-99	-44
Number of IMD deaths		100	-61	-61	-55	-31
Number of long-term survivors with sequelae		72	-40	-40	-37	-17
Total QALYs lost		3,413	-2,044^*^	-2,035^*^	-1,860^*^	-984^*^
Cost of vaccination (2023 USD)		0	1,468.9 M	1,082.7 M	642.1 M	440.6 M
Cost of IMD (2023 USD)		53.6 M	-30.4 M	-30.2 M	-27.8 M	-13.8 M
Short-term medical cost (2023 USD)		47.0 M	-26.7 M	-26.5 M	-24.4 M	-12.0 M
IMD prophylaxis (2023 USD)		6.6 M	-3.8 M	-3.7 M	-3.4 M	-1.7 M
Cost of sequalae (2023 USD)		31.2 M	-17.6 M	-17.4 M	-16.1 M	-7.6 M
Total direct medical costs (2023 USD)		84.8 M	-48.0 M	-47.7 M	-43.9 M	-21.4 M
Total direct costs (2023 USD)		84.8 M	1,421.0 M	1,035.0 M	598.2 M	419.2 M
Indirect costs (2023 USD)		238.3 M	-142.5 M	-141.9 M	-128.9 M	-72.9 M
TOTAL costs (2023 USD)		323.1 M	1,278.4 M	893.1 M	469.3 M	346.4 M
ICER per QALY gained (2023 USD)		-	625,322	438,948	252,249	352,169

Abbreviations: IMD, invasive meningococcal disease; M, million; QALY, quality-adjusted life year

*Negative values represent QALYs gained

**Table 4:** Scenario analysis results

		Incremental vs. No vaccination		
		Q-Q with herd effect (entire US population)	*N*-Q high VCR	Q-*P*-B
Number of IMD cases	Total	-631	-156	-280
	Serogroup A	0	0	0
	Serogroup B	0	0	-5
	Serogroup C	-369	-99	-166
	Serogroup W	-4	-2	-2
	Serogroup Y	-257	-56	-107
Number of IMD deaths		-143	-38	-61
Number of long-term survivors with sequelae		-90	-22	-40
Total QALYs lost		-3,711	-1,229	-2,059
Cost of vaccination (2023 USD)		1,082.7 M	550.8 M	1,561.1 M
Cost of IMD (2023 USD)		-69.3 M	-17.2 M	-30.7 M
Cost of sequalae (2023 USD)		-39.4 M	-9.5 M	-17.7 M
Total direct medical costs (2023 USD)		-108.7 M	-26.7 M	-48.5 M
Total direct costs (2023 USD)		974.0 M	524.0 M	1,512.6 M
Indirect costs (2023 USD)		-267.8 M	-91.1 M	-143.6 M
TOTAL costs (2023 USD)		705.2 M	433.0 M	1,369.1 M
ICER per QALY gained (2023 USD)		190,030	352,169	664,767

Abbreviations: IMD, invasive meningococcal disease; M, million; QALY, quality-adjusted life year

### Estimated IMD burden in the absence of vaccination

Without vaccination, the model estimated that there would be 488 IMD cases per year among the 65.6 million individuals aged 11–25 years in the US. Among the IMD cases, 99% were caused by three serogroups collectively: B (110 cases, 22%), C (231 cases, 47%), and Y (145 cases, 30%). Each year, our model estimated that 100 deaths due to IMD would occur, while 72 individuals would survive with long-term sequelae. The total number of life years (LYs) lost due to IMD in the absence of vaccination was estimated at 2,757 and the total number of QALYs lost, at 3,413. IMD represented a total cost to society of $394 million, including approximately $53.6 million for the treatment of the acute phase and public health response (prophylaxis), $31.2 million for lifetime management of long-term sequelae, and $238.3 million in lifetime indirect costs (including $207.9 million due to premature death).

### Cost-effectiveness of the current vaccination schedule

The current vaccination schedule (Q-QB-B) that includes two doses of MenACWY and two doses of MenB was estimated to avoid 277 IMD cases per year, corresponding to a 57% reduction in IMD cases relative to no vaccination. Of the cases avoided, two were due to the B serogroup (reduction of approximately 2%), and 275 due to C, W, and Y serogroups (73% reduction). The current schedule resulted in a 61% reduction in IMD-related deaths (61 deaths avoided) and a 56% reduction in the number of survivors with long-term sequelae (40 cases avoided). A total of 1,690 LYs and 2,044 QALYs were saved with the use of the current vaccination schedule, representing a reduction of 61% in the number of LYs and 60% in the number of QALYs lost due to IMD without vaccination. Direct medical cost offsets associated with the current vaccination schedule were estimated at approximately $48 million, while indirect cost offsets were higher at $142.5 million, representing a cost reduction of 57% and 59%, respectively, relative to no vaccination. Vaccination costs amounted to $1,468.9 million. Total costs (including direct costs of vaccination, direct medical costs related to IMD and its sequelae, and indirect costs) increased by $1,278.4 million with the current vaccination schedule compared to no vaccination. The ICER for Q-QB-B relative to no vaccination was estimated at $625,322 per QALY (Fig. 2).

### Cost-effectiveness of different vaccination schedules utilizing the quadrivalent meningococcal vaccine

The Q-Q schedule, in which two doses of MenACWY were given at 11–12 and 16 years of age (as per the current schedule) resulted in 275 IMD cases, 61 deaths, and 40 cases with long-term sequelae avoided relative to no vaccination. Consequently, 1,683 LYs and 2,035 QALYs were saved with this schedule relative to no vaccination. Vaccination costs associated with the Q-Q schedule amounted to $1,082.7 million; however, at the same time this schedule produced savings of $47.7 million in direct medical costs and $141.9 million in indirect costs relative to no vaccination. Total incremental costs for the Q-Q schedule were estimated at $893.1 million and the ICER vs. no vaccination was estimated at $438,948 per QALY gained (Fig. 2).

The administration of only a first dose of MenACWY at 11–12 years of age without the booster dose at age 16 years (a Q-N schedule) resulted in 253 cases of IMD avoided, and the prevention of 37 cases of long-term sequelae and 55 IMD-related deaths, relative to no vaccination. Total incremental costs associated with to the Q-N schedule were $469.3 million, resulting in an ICER of $252,249 per QALY gained (Fig. 2).

A single dose of MenACWY administered at 16 years of age, i.e., aligned with the timing of the second dose of the currently administered program (an N-Q schedule), avoided 125 cases of IMD, 31 IMD-related deaths, 17 cases of long-term sequelae, and resulted in an ICER of $352,169 per QALY vs. no vaccination (Fig. 2).

### Scenario analyses

Results of the additional scenarios are presented in Table 4. In the scenario exploring herd immunity with two doses of MenACWY, 631 IMD cases were avoided in the overall population compared to no vaccination. Inclusion of herd immunity resulted in the prevention of 143 deaths and 90 cases of long-term sequelae across the US population. A total of 3,222 LYs and 3,711 QALYs were gained across the overall US population when considering indirect protection, along with $108.7 million and $268.8 million in savings in direct medical costs and indirect costs, respectively. Total incremental cost of the Q-Q schedule was $705.2 million and the ICER relative to no vaccination was $190,030 per QALY gained when herd immunity was considered (Fig. 2).

**Fig. 3:**
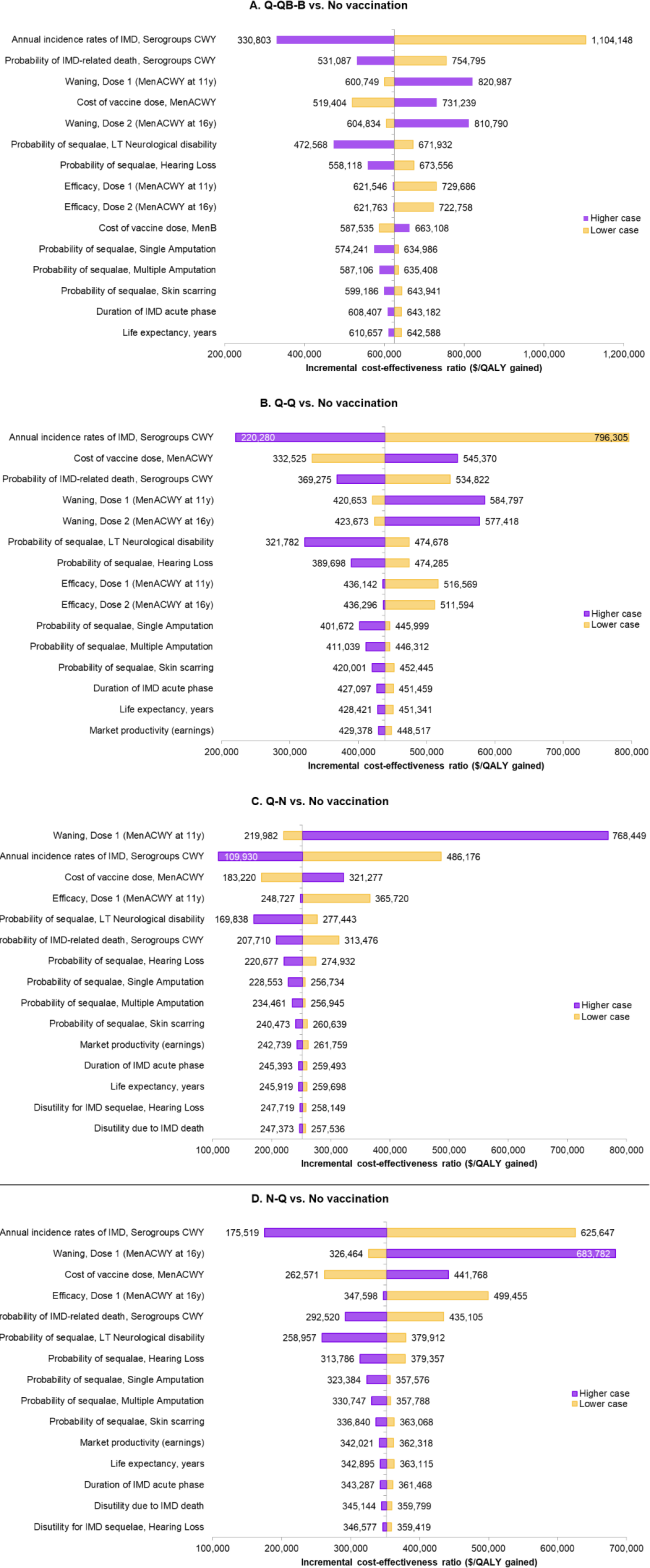
Tornado charts: most 15 impactful parameters of the ICER. Abbreviations: IMD, invasive meningococcal disease; LT, long-term; QALY, quality-adjusted life-year; y, years

In a scenario in which only the MenACWY dose given at 16 years of age was administered and the vaccination coverage rate was hypothetically increased to 76%, 156 cases of IMD were avoided, 38 lives saved, and 22 cases of long-term sequelae prevented. The ICER relative to no vaccination was $352,169 per QALY gained and was therefore the same as the ICER for the N-Q schedule estimated in the base case analysis when current vaccination coverage rates were taken into account (Fig. 3).

In a scenario in which MenABCWY replaced the second dose of MenACWY and the first dose of MenB at age 16 (a Q-P-B schedule), 280 IMD cases (of which 275 were due to serogroups C, W, or Y), 61 deaths, and 40 cases with long-term sequelae were prevented, and 1,700 LYs and 2,059 QALYs were saved compared to no vaccination. Total savings in direct medical costs amounted to $48.5 million, and those in indirect costs to $143.6 million. The annual budget associated with vaccine purchase and administration was estimated at $1,561.1 million with this schedule, while total incremental costs of the schedule were estimated at $1,369.1 million. The ICER compared to no vaccination was estimated at $664,767 (Fig. 2).

**Fig. 4:**
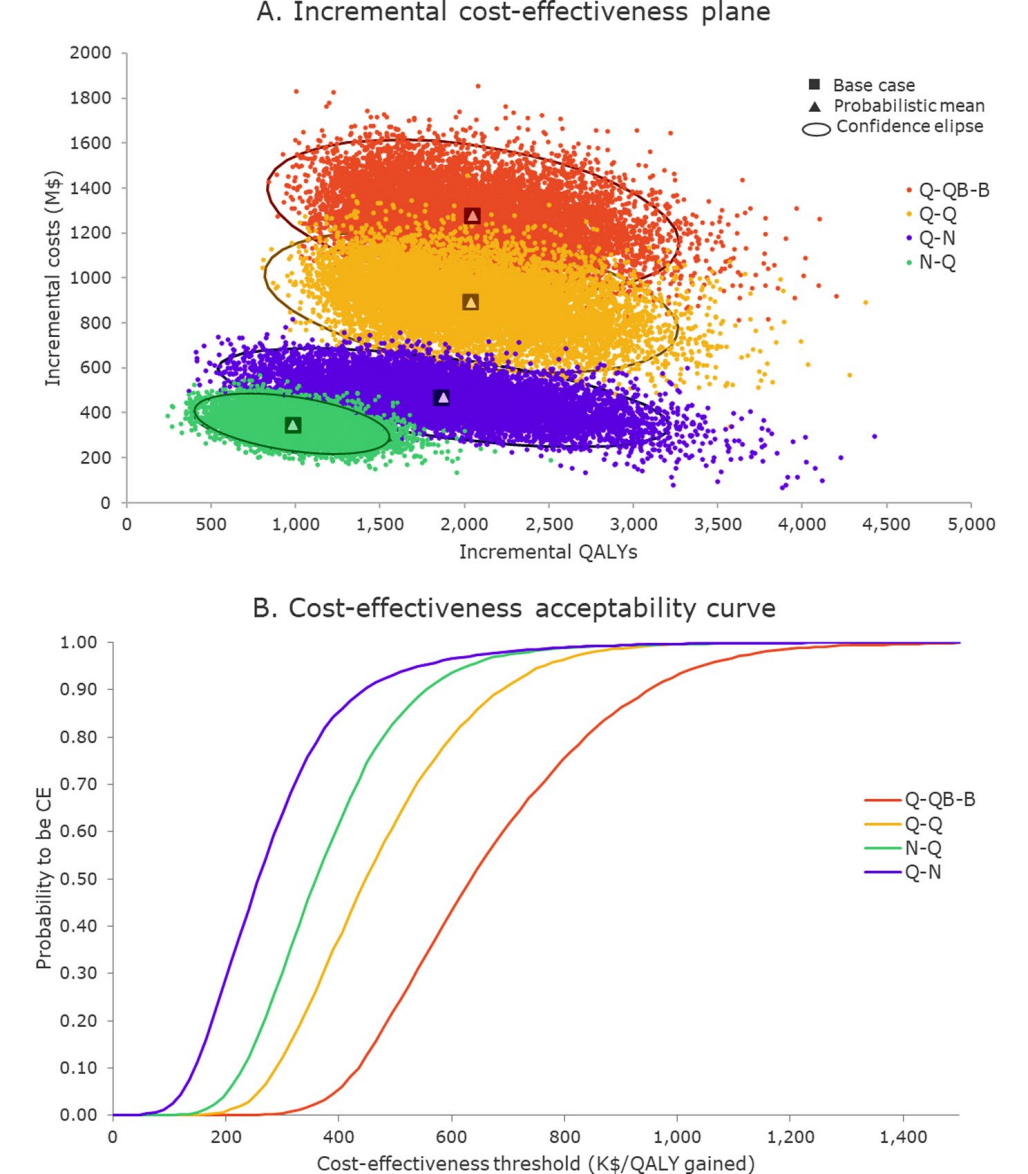
Incremental cost-effectiveness plane (**A**) and cost-effectiveness acceptability curve (**B**) for different vaccination schedules compared to “No vaccination”. Abbreviations: CE, cost-effective; QALY, quality-adjusted life-year

### Sensitivity analyses

In the DSA, ICERs ranged from $330,803 to $1,104,148 for Q-QB-B, from $220,280 to $796,305 for Q-Q, from $109,930 to $768,449 for Q-N, and from $175,519 to $683,782 for N-Q.

As shown in the Tornado diagrams (Fig. 3 and Figure S1–S55) presenting the 15 most impactful parameters among more than 40 tested, the key drivers of the results of the model were similar across all studied schedules, with only the order of their impact changing. The waning rate of protection against serogroups A, C, W, and Y was the most influential parameter affecting the ICER in the Q-N schedule, while annual incidence rates of IMD due to serogroups C, W, and Y were the parameters that had the most impact on other schedules evaluated. In addition to the incidence of IMD, the case-fatality rate and the probability of long-term neurological disability were the most significant clinical determinants of the ICER and of incremental costs and QALYs. Among vaccine-related parameters, waning rates had consistently more impact than did vaccine efficacy. In the schedules assessing a single dose of MenACWY (i.e., Q-N and N-Q), vaccine efficacy was the fourth most influential driver of the ICER and the fifth most influential driver of incremental QALYs. VCR was identified as a driver of incremental costs, but not of incremental QALYs or the ICER. For all schedules and scenarios, the cost of the quadrivalent vaccine was the key driver of incremental costs; for the Q-P-B schedule, the cost of MenABCWY was the second most influential parameter.

As demonstrated by the PSAs (see Fig. 4 and Figure S6), the outcomes of all scenarios were robust to the variability of model parameters. All simulations were in the same quadrant of the incremental cost-effectiveness plane as the base case, except for very few outliers in the Q-Q scenario with herd effect, where this vaccination schedule was dominant. Additionally, mean probabilistic results were very close to deterministic results for all schedules and scenarios.

## Discussion

Without meningococcal vaccination, the model estimated the occurrence of approximately 500 cases of IMD per year in a population of approximately 65 million AYA in the US, 78% of these cases being due to *N. meningitidis* serogroups C, W, and Y. With 100 IMD-related deaths (assuming a 20.5% case-fatality rate) and 72 survivors living with long term sequelae (approximately 15%) per year this represents a significant public health burden. The economic burden that would result is also significant. Direct medical costs would amount to $84.8 million (including $53.6 million spent each year on short-term medical costs and prophylaxis) and a loss in productivity is estimated at $238.3 million, for a total cost of $323.1 million.

The current vaccination program is estimated to have reduced the total number of cases by about 57%, corresponding to a 73% reduction in cases due to serogroups C, W, and Y and 1.8% reduction in cases due to serogroup B. The current vaccination schedule also reduced direct medical costs by 57% and in indirect costs by 60%.

The first dose of MenACWY administered at 11–12 years of age was instrumental in the reduction in IMD burden. Due to the high coverage rate for the dose at age 11–12 and a longer duration of protection for MenACWY, this dose was responsible for a reduction in the number of cases of IMD by almost 52% vs. no vaccination, while two doses of MenACWY produced a reduction of approximately 57%. Conversely, delaying the first dose of MenACWY until 16 years of age (i.e., N-Q) resulted in an approximately 26% reduction in the burden of IMD relative to no vaccination, which represents approximately half of the cases avoided with two doses of the vaccine. Recent and ongoing outbreaks of IMD due to serogroups C and Y in the US demonstrate that *N. meningitidis* continues to circulate [7]. Given the importance of the first dose administered at 11–12 years of age for the prevention of IMD demonstrated by our analysis, discontinuation of this vaccination could result in an increase in cases akin to that seen with other vaccine-preventable diseases after a reduction in vaccination coverage [38]. Additionally, the DSA showed that Q-N was less sensitive to variations in incidences than N-Q, especially in scenarios with lower incidences. This tends to demonstrate that the benefit of a single dose given at 11–12 years (Q-N) is more stable than a single dose administered at 16 years (N-Q).

The high vaccination coverage rates observed with the first dose of MenACWY at age 11–12 years likely reflect the fact that this vaccination is required for school entry in 36 states (vs. 25 states for the booster dose at 16 years of age) [39]. If health authorities wish to preserve the current effectiveness of their immunization strategy, they may seek to further increase coverage rates, especially for older adolescents. However, even if the coverage rate for a single dose administered at age 16 were > 75%, this single-dose strategy (i.e., N-Q with 25% increase in coverage rate) would not be as effective as two doses or a single dose administered at 11 years of age. Furthermore, increasing vaccination coverage among 16-year-olds may not be feasible, as suggested by the observed decreases in compliance with well exams as adolescents age and the 14 years of attempts to raise these rates in the US [40–42].

In European countries, where priming vaccination primarily targets infants, meningococcal vaccination schedules have recently evolved to include a booster dose of MenACWY for adolescents (11–18 years) [43]. Removing the first dose of MenACWY would contradict with the current trend of strengthening protection for exposed and vulnerable populations, particularly adolescents, who face an increased risk of meningococcal disease. On the contrary, maintaining the first dose of MenACWY would align with the broader public health objective of extending immunity during critical periods of susceptibility, thereby reducing the burden of disease and preventing outbreaks in this age group.

In the scenario incorporating herd protection in the overall US population, the Q-Q vaccination schedule prevented 2.3 times more IMD cases than when considering AYA alone in the base case analysis, resulting in additional savings of $61 million in direct medical costs and $126 million in indirect costs. With the inclusion of herd immunity, the ICER associated with the Q-Q schedule would fall by 56% relative to the base case. The herd immunity scenario was based on real-world evidence that demonstrated a significant reduction in both meningococcal carriage in adolescents [44] and in the number of cases in unvaccinated individuals following the introduction of a vaccination program, against serogroup C in the UK [29]. With a two-dose vaccination program in place for about nine years and high coverage rates, it is reasonable to expect that herd immunity is effective in the US, and a similar hypothesis was previously considered in several modelling exercises conducted in the US [33, 45] and elsewhere [46–49]. Our assumption regarding the level of herd protection was even more conservative when compared to previous analyses conducted by Ortega-Sanchez et al. [33], where the level of indirect protection considered was more than two times higher than was assumed in our study. Additional research should be conducted in the US to better understand the role of vaccination in inferring indirect protection. Nevertheless, if the first dose of MenACWY is removed, from the age group with the highest vaccination coverage rates, the level of herd protection might be reduced, and an increase in the number of IMD cases could be observed in all age groups.

The administration of the MenB vaccine is currently guided by a shared clinical decision-making process, which places the decision to vaccinate largely on individual considerations rather than universal recommendations. Because of this approach, vaccination coverage rates are 29.4% for individuals who received at least one dose of MenB vaccine and 11.9% for those who completed the two-dose regimen as of 2022 [18]. These coverage rates impact the vaccine’s public health utility, as the MenB is considered to provide short-term duration of individual protection only after the complete two-dose course, which achieves an efficacy rate of 85% [26, 31]. These factors collectively limited the effectiveness of the current vaccination strategy in preventing meningococcal B disease. The low vaccination rates coupled with the necessity of completing the full regimen resulted in relatively few cases averted. This analysis underscores the importance of enhancing vaccination uptake for the second dose. Substantial gains in population-level protection and disease prevention could be realized by increasing the proportion of individuals receiving the full two-dose series.

The incremental gains associated with the administration of MenABCWY (Q-P-B) were estimated to be relatively limited, with only 3 additional IMD cases due to serogroup B avoided at a 6% increase in immunization costs. A Q-P-B schedule, compared to no vaccination, was estimated not to be cost-saving, with an ICER greater than that of the vaccination schedule currently in place. When indirectly compared to Q-QB-B (i.e. Q-P-B vs. no vaccination compared to Q-QB-B vs. no vaccination), our findings for Q-P-B are relatively similar to the CDC and Pfizer analyses presented in October 2023 [31] in terms of humanistic burden avoided; however, there is a substantial difference in the estimate of incremental costs. While we found Q-P-B to be associated with additional costs, CDC and Pfizer found it to be a cost saving schedule compared to Q-QB-B. This is certainly due to the fact that CDC and Pfizer analyses compared schedules without considering the current vaccination coverage rates (i.e. all individuals entering the model were assumed to be vaccinated), while our model reflected actual coverage rates observed in the US. In the Q-P-B scenario, we assumed that all adolescents who currently receive the second dose of MenACWY will switch to a dose of MenABCWY, maintaining a VCR of 60%; this assumption leads to an increase of the vaccination costs (versus Q-QB-B) which is not entirely compensated by the reduction in IMD costs.

Our results pertaining to IMD burden are comparable to those from the CDC analyses presented to the ACIP in June 2023 [26], although a larger number of premature deaths was estimated by our analysis, which considered mortality post-discharge. The inclusion of post-discharge mortality resulted in lower ICERs, with the ICER for the current vaccination schedule ($625,322 per QALY) and the Q-Q schedule ($438,948 per QALY) being lower than that estimated by the CDC ($1,498,000 per QALY for Q-QB-B and $550,000 for Q-Q) [26]. Of note, we used a less conservative assumption regarding the duration of protection for MenACWY than was considered in recent analyses [31, 45]. Nevertheless, this assumption was based on the most recent evidence from a clinical trial showing a high level of seroprotection in adolescents up to 6 years after receipt of a first dose of the vaccine [17]. More long-term data would be necessary to better estimate the vaccine duration of protection and reduce the associated uncertainty.

The results of our analysis assessing a single dose given at 11–12 years of age were similar to those reported by Shepard et al. [27] in terms of the number of IMD cases avoided (253 vs. 270) and reduction in societal costs of IMD (53% vs. 46%). However, it should be noted that the two studies used a different approach to model the vaccinated population. While Shepard followed a population of 11-year-olds over 22 years [27], the current model included a cross-sectional population of American AYA aged 11–25 years in a given calendar year.

The strength of this analysis lies in using well-recognized and most recent public health data sources, similar to those used in recent CDC analyses [26] and supplementing them with relevant literature. Furthermore, with probabilistic ICERs very close to base cases, the results were robust to the observed variability of inputs, model parameter distributions were mostly sourced from literature. Comparing all vaccination schedules to the “No vaccination” situation can help decision makers fully understand the value of the different components of the current schedule, as well as alternative vaccination schedules. Additional scenarios were evaluated for each comparison of vaccination schedules, incorporating variations in incidence rates (high vs. low) and duration of protection (long vs. short). This analysis was conducted to further assess the consistency and robustness of the results beyond the initial sensitivity analyses. The findings demonstrated that the outcomes remained consistent and robust across a range of input assumptions. Detailed results are available upon request. Overall, this analysis is well-equipped to inform on the real value of the current US meningococcal vaccination program.

Limitations of the current analysis include the conservative modelling approach, in which the impact of IMD on the quality of life of family and caregivers were not included, despite an analysis of infant vaccination against serogroup B in England highlighting the importance of these factors for accurately evaluating IMD burden and cost-effectiveness of vaccination [50]. Moreover, this cost-utility analysis does not consider the equity dimension. The literature shows that inequalities exist in the access to IMD prevention, with lower socioeconomic levels associated with lower VCR, as well as a lower vaccine uptake observed among individuals covered by Medicaid compared to those covered by private or commercial insurance [51–53]. Additionally, racial and ethnic minorities, as well as those from low-income families, often have higher rates of meningococcal disease [53]. Eliminating the first dose of MenACWY at 11–12 years could disproportionately affect vulnerable populations and leave some at-risk groups unprotected during a critical period. Furthermore, the current two-dose schedule helps ensure more equitable protection across all socioeconomic groups, as school-entry requirements often increase compliance.

Significant barriers to equitable meningococcal vaccine access persist among U.S. adolescents and young adults, including limited provider recommendations, low disease and vaccine awareness, inadequate preventive care visits, financial constraints, as well as the absence of school entry requirements in many states, which can particularly impact MenACWY booster coverage [51, 53]. To address these disparities, targeted interventions such as expanding vaccine availability through school-based or community health programs, reducing financial burdens via enhanced insurance coverage or vaccine subsidies, and implementing culturally tailored education campaigns could be instrumental.

In the context of rising antimicrobial resistance (AMR), with Y serogroup IMD cases resistant to ciprofloxacin and β-lactam antibiotics reported in the US [7, 54, 55], the benefits of meningococcal vaccination could be higher than assessed in this study. By preventing infections, vaccination could reduce the healthcare costs associated with treating resistant infections, which often require longer hospital stays and more expensive antibiotics. By limiting the number of infections, meningococcal vaccination could also reduce the use of antibiotics and thus potentially slow the development of AMR. The World Health Organization (WHO) estimated that existing vaccines globally could reduce antibiotic use by 142 million defined daily doses (DDDs) [56]. Additionally, its role in protecting vulnerable populations (e.g. immunocompromised individuals), relying on both, vaccination and antibiotic prophylaxis, could be strengthen. Including these two dimensions in future research should provide more accurate estimates of the value of IMD vaccination in the US.

There exists some uncertainty regarding estimated IMD incidence, given that pre-vaccination era (i.e., greater than 20 years old) data were used to estimate IMD incidence in the absence of vaccination. Furthermore, our model was unable to account for the natural fluctuation in the circulation of *N. meningitidis* and did not include disease outbreaks. Additionally, compared to dynamic modelling, we used a more simplistic approach for considering the herd effect. Finally, the model considered the steady state of vaccination schedules, without including the impact of schedule changes on effective coverage rate.

## Conclusion

The currently recommended schedule for MenACWY vaccination in the US plays a crucial role in reducing the burden of IMD caused by commonly circulating meningococcal serogroups in the US. The first dose, administered at 11–12 years of age, contributes significantly to this reduction. Any updates to the adolescent meningococcal vaccination schedule in the US should weigh the potential cost savings against the risk of premature mortality and long-term disability associated with IMD.

## Electronic supplementary material

Below is the link to the electronic supplementary material.

Supplementary Material 1: Supplementary Methods, Table S1 (Base case value and sensitivity analysis inputs of main model parameters) and Figures S1 to S6 (Tornado diagrams and Incremental cost-effectiveness planes and cost-effectiveness acceptability curves for scenarios) are available in the associated “Supplementary material 1.docx” file.

## Data Availability

No datasets were generated or analysed during the current study.

## Abbreviations

ACIP: Advisory Committee on Immunization Practices
AYA: Adolescents and young adults
CDC: Centers for Disease Control and Prevention
DSA: Deterministic sensitivity analysis
IMD: Invasive meningococcal disease
ICER: Incremental cost-effectiveness ratio
LY: Life-year
PSA: Probabilistic sensitivity analysis
QALY: Quality-adjusted life year
UK: United Kingdome
US: United States
VCR: Vaccination coverage rate
