# Supplementary material for: Understanding the value of meningococcal vaccination for adolescents and young adults in the United States: insights from a steady-state modelling approach
Source: BMC Public Health. 2025 May 20;25:1863. doi: 10.1186/s12889-025-21953-8 (PMC12090439; doi:10.1186/s12889-025-21953-8)
Supplement: Supplementary file 1 — Supplementary Material 1: Supplementary Methods, Table S1 (Base case value and sensitivity analysis inputs of main model parameters) and Figures S1 to S6 (Tornado diagrams and Incremental cost-effectiveness planes and cost-effectiveness acceptability curves for scenarios) are available in the associated “Supplementary material 1.docx” file. [file 12889_2025_21953_MOESM1_ESM.docx]

# Understanding the value of meningococcal vaccination for adolescents and young adults in the United States: insights from a steady-state modelling approach

Langevin E^1^, Robertson C^2^, Galarza K^2^, Dogu A^3^, Cristeau O^4^, Clay E^4^, Wu J^5^, Shin T^5,6^

# Supplementary Methods

## Population

This cost-effectiveness analysis focused on a cohort of US adolescents and young adults (AYA) aged 11–25 years, analyzed in two age-based subgroups, 11–17 years and 18–25 years. The age of the modelled cohort covered the 15-year age span benefiting the most from the direct protection brought by the current US adolescent meningococcal vaccination program and was also in line with the most recent analysis conducted by the Centers for Disease Control and Prevention (CDC) and presented to the Advisory Committee on Immunization Practices (ACIP) [1, 2].

In addition to assessing the cost-effectiveness of meningococcal vaccination in the 11–17 and 18–25 year age groups, an analysis evaluated the value of vaccination when the US population was further stratified into six age groups (i.e. 0–10, 11–17, 18–25, 26–32, 33–64, and ≥65). This was done to support the scenario analysis examining the indirect effect of vaccination (i.e., herd immunity).

The most recent demographic data available from official sources were used. Population size by year of age for the 2022 calendar year was retrieved from the US Census Bureau to calculate the population size of age groups of interest [3]. The percentage of females was estimated from the 2022 American Community Survey data available on the US Census Bureau website [4]. The 2021 life expectancy at birth for males and females was used to estimate life years (LYs) lost; this was retrieved from the life tables published by the CDC in its National Vital Statistics Reports [5].

## Vaccination schedules

Vaccination schedules were defined as a series of doses. For each dose administered, the model considered the type of meningococcal vaccine (monovalent serogroup B vaccine [MenB], quadrivalent vaccine [MenACWY], or pentavalent vaccine [MenABCWY]), the age at vaccination, the vaccination coverage rate (VCR), the serogroup-dependent vaccine effectiveness and the rate of its waning. Based on these parameters, the model estimated the level of direct vaccine protection against each *N. meningitidis* serogroup in each year of age of individuals in the cohort. The weighted average level of protection was then computed by serogroup for each age group. Vaccine protection represented the reduction in number of invasive meningococcal disease (IMD) cases with vaccination.

## Vaccine efficacy

The assumption of 97% of vaccine effectiveness for MenACWY was based on seroprotection data from two recent phase 2 and phase 3 randomized clinical trials (RCT) for MenACWY-TT (MenQuadfi^®^, Sanofi Pasteur Inc.) [6, 7]. These data were expressed as the proportion of adolescent participants (10-17 years of age) with seroprotection measured 30 days after the administration of a single vaccine dose. Seroprotection was determined using a human complement serum bactericidal assay (hSBA; Global Clinical Immunology, Sanofi, Swiftwater, PA, USA). In both trials, seroprotection was defined as hSBA titers ≥ 1:8. In the phase 2 RCT, hSBA seroprotection rates varied between 93.5% for serogroup A and 99.1% for serogroup W, with an average across the four serogroups of 97% [6]. Seroprotection rates measured in the phase 3 trial ranged between 96.2% for serogroup A and 99.1% for serogroup Y, with an average across the four serogroups of 98% [7]. We used the most conservative value between the average seroprotection rates of both trials. The seroprotection rate for serogroup C was 98.5% in both trials, while MenC is the most represented among IMD cases due to ACWY serogroups in US AYA. Our assumption is therefore conservative.

The waning rate was estimated based on data from a phase 3b open-label trial where 82.5% (average across the four serogroups) of participants were still seroprotected (i.e. hSBA titers ≥ 1:8) 3–6 years after having received a priming dose of MenACYW-TT [8]. The simplest assumption of linear decay of the seroprotection was assumed to compute the annual rate. Assuming that the remaining seroprotection was observed at 4.5 years (mid-point of the observation range) the waning rate was estimated as follows:

$$\frac{Seroprotection at 30 days-seroprotection at 4.5 years}{4.5 years}=\frac{97\%-82.5\%}{4.5} \approx3\%$$

## Modelling IMD burden

The model predicted the number of IMD cases occurring over one year with each vaccination schedule based on IMD incidence by age and serogroup and the level of protection obtained through vaccination. To accurately reflect the reduced quality of life (QoL) of individuals developing IMD and the associated costs, IMD cases were stratified by clinical presentation including meningitis, septicemia, and other manifestations of meningococcal infection. Each type of presentation was associated with a hospitalization cost and a disutility applied for a certain duration. The costs of the public health response were also considered for each IMD case occurring in the model.

Both short-term and long-term mortality associated with IMD were considered. Deaths occurring during the acute phase were estimated from the case-fatality rate (CFR) by age and serogroup. Long-term mortality was modelled among the survivors of the acute phase. An incidence rate ratio (IRR) was applied to the CFR to estimate mortality rates reflecting that a proportion of IMD-related mortality occurs post discharge. Quality-adjusted life-years (QALYs) lost due to IMD-related premature death were estimated over a lifetime horizon.

Morbidity of IMD was considered through long-term sequelae, which included skin scarring, single and multiple amputation, hearing loss, and significant long-term neurologic disability (as in the CDC model). Costs and QALYs lost associated with long-term sequelae were estimated over the patients’ lifetime. The model also considered reduced QoL after the acute phase for IMD survivors without long-term sequelae.

Indirect costs were estimated over a lifetime horizon considering patient’s productivity losses related to long-term sequelae and premature death, and the productivity losses for caregivers associated with the acute infection phase.

## Model outcomes

For each schedule, the model estimated health and cost outcomes. Health outcomes included the number of IMD cases (by serogroup), the number of IMD-related deaths, the number of long-term survivors with sequelae, and total number of QALYs lost. Costs included direct and indirect costs. Total direct costs included vaccination costs as well as total IMD-related costs (short-term medical costs and prophylaxis of close contacts) and the costs of managing sequelae, which together comprised total direct medical costs. Incremental results and incremental cost-effectiveness ratio (ICER) were computed relative to no vaccination for each vaccination schedule.

## Clinical inputs

Model inputs are summarized in Table S1. IMD incidence was sourced from publicly available US surveillance data collected prior to the implementation of the meningococcal vaccination program (1996–2005 for CWY and 1994–2013 for B) [1], and attributed to specific serogroups based on the study by Shepard et al [9]. Subsequently, weighted average incidence was computed by age group based on historical (1993–2002) US population demographics [3], fitting to Shepard et al. epidemiological data [9] to obtain higher age granularity of incidence data by serogroup.

Case-fatality rates for serogroups B and CWY from the National Notifiable Diseases Surveillance System (NNDSS) for 2008–2020 were used in the model [10]. Data were adjusted to the age groups considered in our study using population data from the US Census Bureau for the same years to compute a weighted average [3]. The ratio of in-hospital to post-discharge mortality for patients with and without sequelae was informed by the study by Shen et al [11]. Thus, the model assumed that a third of IMD-related deaths occurs three years after discharge, in line with Shen et al [11]. Probabilities of sequelae were based on the studies by Shepard and Ortega-Sanchez [1, 9].

## Utilities

The analyses included only QALYs lost by patients; caregiver utility was not considered. QALYs lost were discounted at a 3% annual rate, similar to previous health economic analyses and in line with recommendations for US economic evaluations [1, 2, 12, 13].

Baseline utility for US individuals aged <25 years was sourced from Jiang et al. [14] and used to estimate the lifetime QALYs lost due to premature death. Utility decrements associated with the acute phase of IMD, stratified by clinical presentation, were sourced from Lecoq et al. [15] and were applied during the acute phase lasting for the first year post IMD, in line with an industry analysis presented to the ACIP in October 2023 [2].

Consistent with the same analysis presented to the ACIP, a disutility of 0.03 [16, 17] was applied for 9 years after the acute phase for patients with IMD who had no permanent sequelae. For individuals with long-term sequelae, utility decrements were derived from the CDC analyses and utilities previously used by Ortega-Sanchez [1, 9, 18]. These utilities were first adjusted to the baseline utility for AYA (Ortega-Sanchez assumed a baseline utility of 1 in his study), and then subtracted from the baseline utility.

## Costs and resource use

Costs were expressed in 2023 USD. Literature-derived historical costs were inflated to 2023 values using the average Consumer Price Index (CPI) for All Urban Consumers in US City. Medical costs were inflated using the CPI for medical care, while productivity was inflated using the CPI for all items. Costs were discounted at an annual rate of 3%, similar to previous health-economic analyses and in line with recommendations for US economic evaluations [1, 2, 12, 13].

Unit costs per type of IMD clinical presentation, including acute hospitalization and post discharge follow-up care costs, were obtained from Davis et al [19]. Discounted lifetime costs per IMD sequelae used in the CDC analyses presented to the ACIP in June 2023 and initially estimated by Shepard and Ortega-Sanchez were considered [1, 9, 18]. In addition to medical care, amputation costs included prosthesis and rehabilitation costs. For long-term neurological disability, costs of special education needs were included for cases occurring before the individual was 18 years of age.

The approach to modelling indirect costs associated with lost productivity followed the CDC analyses [1, 2]. The value of work time lost by caregivers during the acute phase of IMD was sourced from the CDC analyses presented to the ACIP in June 2023 [1]. Yearly productivity by age in the USA estimated by Grosse et al. [20] was used to estimate long-term productivity losses. Lifetime productivity lost due to premature death included labor market productivity (i.e., earnings) and non-labor market productivity (i.e., household services consumption). Data from Grosse [20] were adjusted to the age groups considered in our study based on the 2022 US population age structure [3]. For productivity losses associated with long-term sequelae of IMD, we assumed the same proportions of market productivity lost as Ortega-Sanchez, i.e., from 20% for single amputation to 100% for long-term neurologic disability [18].

Average vaccination cost per dose was estimated for each vaccine type in both, the private and public sector, considering acquisition, wastage, and administration costs. The same assumptions as in the CDC analyses were used for the proportions of vaccines purchased (54%) and administered (22%) in the public sector, as well as for the percentage of wasted doses (4.5%). Vaccine costs per dose were based on most recent publicly available data for the public and private sector [21]. The price of MenQuadfi^TM^ was assumed for MenACWY and the price of Bexsero^®^ was assumed for MenB. For administration costs, the same costs as in the CDC analyses were used ($15 for doses administered in the public sector and $30 in the private sector) [1].

## Sensitivity and scenario analyses

Univariate deterministic sensitivity analyses (DSAs) were conducted on all model parameters associated with uncertainty (see Table S1) by changing the value of one model parameter or one assumption at a time. Parameter ranges for the sensitivity analyses were obtained using credibility or confidence intervals, standard deviations, fixed values from the literature or, when none of these were available, a ± 20 % variation from the base case value. Main model outcomes (number of IMD cases avoided, incremental QALYs, incremental costs, and ICER) were computed using the low and high values of each model parameter.

Probabilistic sensitivity analysis (PSA) was performed to address parameter uncertainty and assess the robustness of our deterministic results. Appropriate statistical distributions were assigned to each of the model parameters; these are listed in Table S1. When not available from the literature, standard deviations were calibrated to obtain a 95% credibility interval that is as close as possible to the DSA range. For parameters which value differs by age group, a factor following the appropriate distribution (e.g., Beta for probabilities, Gamma for costs) was simulated, and values for each age group were multiplied by this factor. This ensured that the relative risks observed between age groups were maintained and avoided the simulation of unrealistic scenarios. The model was run 10,000 times with random values drawn from these statistical distributions to obtain the distribution of the model outcomes (ICER, total incremental costs, and QALYs).

# Supplementary Tables

Table S1: Base case value and sensitivity analysis inputs of main model parameters

| **Parameter** | **Parameter characteristics** | **Base case value** | **Range for DSA** | **Distribution for PSA**  **Theoretical 95% CI of simulated values** | **Source** |
| --- | --- | --- | --- | --- | --- |
| Life expectancy (years) | Women | 79.3 | [75.3 ; 83.3] | Normal(μ=79.30, σ=2.02) | CDC National Vital Statistics Reports [5]  Range: assumption base case ±5% |
|  | Men | 73.5 | [69.8 ; 77.2] | Normal(μ=73.50, σ=1.88) |  |
| Proportion of women | | 50.4% | [50.3% ; 50.5%] | Beta(μ=50.4%, σ=0.001) | US Census Bureau [4]  Range: base case ±0.1% (Census bureau) |
| Population size by age group | 11–17 years | 29,878,732 | NA | NA | US Census Bureau [3]  Not included in SA |
|  | 18–25 years | 35,674,439 | NA | NA |  |
| IMD rates | 11–17 years | A: 0.0  B: 0.974  C: 0.3356  W: 0.0  Y: 0.2707 | B: [-19.0% ; +21.0%]  C: [-13.0% ; +14.0%]  W: [-17.0% ; +18.0%]  Y: [-84.0% ; +160.0%] | B: Beta(μ=0.50, σ=0.11)+0.5 => ±21.0%  C: Beta(μ=0.50, σ=0.07)+0.5 => ±14.0%  W: Beta(μ=0.50, σ=0.09)+0.5 => ±18.0%  Y: Beta(μ=0.50, σ=0.47)+0.5 => ±50.0% | US surveillance data [1] and Shepard et al. [9]  Range: % of variation estimated using 95% CI of beta distributions with number of cases and age group size |
|  | 18–25 years | A: 0.0  B: 0.2256  C: 0.3652  W: 0.0079  Y: 0.1809 |  |  |  |
| Distribution of clinical presentation of IMD | Meningitis | 50.6% | [52.5% ; 47.7%] | Dirichlet(α1=1,536.05, α2=1,013.91, α3=487.05) | Davis et al. [19]  Range: 95% CI of a Dirichlet distribution based on number of cases in Davis et al. |
|  | Septicemia | 33.4% | [32.7% ; 34.9%] |  |  |
|  | Other meningococcal infection | 16.0% | [14.8% ; 17.4%] |  |  |
| Probability of occurrence of permanent sequelae | Skin scarring | 7.6% | [0.0% ; 19.0%] | Beta(μ=0.08, σ=0.05) => [1.2% ; 19.0%] | Ortega-Sanchez [1] |
|  | Single amputation | 1.9% | [0.5% ; 10.0%] | Beta(μ=0.02, σ=0.03) => [0.0% ; 10.0%] |  |
|  | Multiple amputation | 1.2% | [0.0% ; 6.0%] | Beta(μ=0.01, σ=0.02) => [0.0% ; 6.0%] |  |
|  | Hearing loss | 8.8% | [2.0% ; 20.0%] | Beta(μ=0.09, σ=0.05) => [2.0% ; 20.0%] | Ortega-Sanchez [1] |
|  | Significant long term neurologic disability | 2.1% | [0.0% ; 11.0%] | Beta(μ=0.02, σ=0.03) => [0.0% ; 11.0%] | Ortega-Sanchez [1] |
| Case-fatality rate in the acute phase of serogroup B disease | 11–17 years | 8.7% | [-50.5% ; +68.5%] | Beta(μ=0.50, σ=0.45)+0.5 => ±50.0% | NNDSS [10]  Range: % of variation estimated using 95% CI of beta distributions based on number of fatal cases and incidences |
|  | 18–25 years | 9.2% | [-50.5% ; +68.5%] | Beta(μ=0.50, σ=0.45)+0.5 => ±50.0% |  |
| Case-fatality rate in the acute phase of serogroup CWY disease | 11–17 years | 12.3% | [-23.2% ; +23.7%] | Beta(μ=0.50, σ=0.12)+0.5 => ±23.7% |  |
|  | 18–25 years | 18.3% | [-23.2% ; +23.7%] | Beta(μ=0.50, σ=0.12)+0.5 => ±23.7% |  |
| Excess mortality in survivors (ratios of post-acute and acute case-fatality rates) | IMD without sequelae | 0.422 | [0.380 ; 0.454] | NA | Shen et al. [11]  Range: 95% CI of Beta distributions based on number of post-acute deaths and number of subjects |
|  | IMD with sequelae | 1.012 | [0.889 ; 1.124] | NA |  |
| Vaccination coverage (MenACWY) | 11 years | 88.6% | [87.6% ; 89.6%] | Beta(μ=0.88, σ=0.01) => [87.6% ; 89.6%] | 2022 National Immunization Survey [22]  Range: 95% CI from [22] |
|  | 16 years | 60.8% | [57.5% ; 63.9%] | Beta(μ=0.61, σ=0.02) => [57.5% ; 64.1%] |  |
| Vaccination coverage (MenB) | 16 years | 29.4% | [26.5% ; 32.4%] | Beta(μ=0.29, σ=0.02) => [26.5% ; 32.4%] |  |
|  | 16.5 years | 11.9% | [10.0% ; 14.1%] | Beta(μ=0.12, σ=0.01) => [9.9% ; 14.1%] |  |
| Vaccination coverage (scenario QPB) | 11 years (MenACWY) | 88.6% | [87.6% ; 89.6%] | Beta(μ=0.89, σ=0.01) => [87.6% ; 89.6%] | 2022 National Immunization Survey [22]  Range: 95% CI from [22] |
|  | 16 years (MenABCWY) | 60.8% | [57.5% ; 63.9%] | Beta(μ=0.61, σ=0.02) => [57.5% ; 64.1%] |  |
|  | 16.5 years (MenB) | 30.4% | [27.4% ; 33.4%] | Beta(μ=0.30, σ=0.02) => [27.5% ; 33.4%] | Assumption 50% of VCR at 16 years  Range: assumption ±10% |
| Vaccine efficacy (first dose / two-dose course) | MenB vaccine | 0.0% / 85.0%^a^ | [18.0% ; 64.0%] / [50.0% ; 99.0%] | Beta(μ=0.60, σ=0.21) => [18.0% ; 94.0%] /  Beta(μ=0.85, σ=0.14) => [50.0% ; 99.7%] | Ortega-Sanchez [1] |
|  | MenACWY | 97.0% / 97.0%^a^ | [73.0% ; 98.0%] | Beta(μ=0.97, σ=0.08) => [73.0% ; 100.0%] | Chang et al. [6] and Dhingra et al. [7]  Range from Ortega-Sanchez [1] |
| Annual waning rate | MenACWY | 3.0% (linear) | [2.0% ; 10.0%] | Beta(μ=0.03, σ=0.03) => [0.1% ; 10.0%] | Assumption based on clinical data [8]  Range: assumptions |
|  | MenB | 33.3% (exponential) | [20.0% ; 50.0%] | Beta(μ=0.33, σ=0.08) => [18.6% ; 50.0%] | Assumptions based Ortega-Sanchez [1] |
| Indirect protection | | 35.0% | [20.0% ; 49.0%] | Beta(μ=0.35, σ=0.08) => [20.0% ; 51.7%] | Ramsay et al. [23]  Range: Ortega-Sanchez [18] |
| Vaccination costs (USD) | MenACWY | 169.78 | [135.82 ; 203.74] | Gamma(μ=169.78, σ=18.30) => [135.82 ; 207.47] | CDC Vaccine Price List [21] and assumptions from Ortega-Sanchez [1]  Range: base case ±20% |
|  | MenB | 219.11 | [175.29 ; 262.93] | Gamma(μ=219.11, σ=23.61) => [175.29 ; 267.74] |  |
|  | MenABCWY | 244.58 | [195.66 ; 293.50] | Gamma(μ=244.58, σ=26.36) => [195.66 ; 298.87] |  |
| Direct costs in the acute phase (hospitalization) (USD) | Meningitis | 81,741.24 | [65,393.00 ; 98,089.49] | Gamma(μ=81,741.24, σ=8,808.36) => [65,392.99 ; 99,886.03] | Davis et al. [19]  Range: base case ±20% |
|  | Septicemia | 115,841.55 | [92,673.24 ; 139,009.86] | Gamma(μ=115,841.55, σ=12,482.97) => [92,673.24 ; 141,555.85] |  |
|  | Other meningococcal infection | 101,269.73 | [81,015.78 ; 121,523.67] | Gamma(μ=101,269.73, σ=10,912.72) => [81,015.78 ; 123,749.40] |  |
| Public health response costs (USD) | | 13,604.56 | [1,798.61 ; 16,076.01] | Gamma(μ=13,604.56, σ=9,348.08) => [1,798.61 ; 37,094.25] | Ortega-Sanchez [2] |
| Direct costs of sequelae^b^ (USD) | Skin scarring | 7,467.59 | [6,878.10 ; 11,200.38] | Gamma(μ=7,467.59, σ=1,721.44) => [4,483.59 ; 11,200.38] | Ortega-Sanchez [1] |
|  | Single amputation | 204,360.57 | [102,179.28 ; 306,539.85] | Gamma(μ=204,360.57, σ=61,496.29) => [102,179.28 ; 341,361.21] |  |
|  | Multiple amputation | 245,234.49 | [122,615.74 ; 367,849.22] | Gamma(μ=245,234.49, σ=73,796.32) => [122,615.74 ; 409,637.02] |  |
|  | Hearing loss | 89,946.53 | [26,301.27 ; 113,922.96] | Gamma(μ=89,946.53, σ=43,097.57) => [26,301.27 ; 192,009.35] |  |
|  | Significant long term neurologic disability – 0–17years | 2,921,074.90 | [1,050,808.55 ; 3,548,393.83] | Gamma(μ=2,921,074.90, σ=1,211,441.26) => [1,050,808.55 ; 5,730,559.75] |  |
|  | Significant long term neurologic disability – ≥18 years | 2,716,194.00 | [926,934.49 ; 3,302,121.95] | Gamma(μ=2,716,194.00, σ=1,172,300.36) => [926,934.49 ; 5,450,188.67] |  |
| Value of work time lost by caregivers in the acute phase (USD) | | 4,298.24 | [3,438.59 ; 5,157.89] | Gamma(μ=4,298.24, σ=463.17) => [3,438.59 ; 5,252.36] | Ortega-Sanchez [1]  Range: base case ±20% |
| Market productivity (earnings) (USD) | 11–17 years | 5,556.44 | [4,445.15 ; 6,667.72] | Gamma(μ=1.00, σ=0.11) => [0.80 ; 1.22] | Grosse et al. [20]  Range: base case ±20% |
|  | 18–25 years | 17,471.37 | [13,977.10 ; 20,965.65] | Gamma(μ=1.00, σ=0.11) => [0.80 ; 1.22] |  |
| Non-market productivity (household services),  applied to IMD death only (USD) | 11-17 years | 5,619.62 | [4,495.69 ; 6,743.54] | Gamma(μ=1.00, σ=0.11) => [0.80 ; 1.22] | Grosse et al. [20]  Range: base case ±20% |
|  | 18-25 years | 15,090.45 | [12,072.36 ; 18,108.54] | Gamma(μ=1.00, σ=0.11) => [0.80 ; 1.22] |  |
| Productivity loss | Skin scarring | 0.0% | [0.0% ; 5.0%] | Beta(μ=0.05, σ=0.02) => [0.0% ; 5.0%] | Ortega-Sanchez et al. [18]  Range: assumption base case ±5 points of % |
|  | Single amputation | 20.0% | [15.0% ; 25.0%] | Beta(μ=0.20, σ=0.03) => [15.0% ; 25.5%] |  |
|  | Multiple amputation | 30.0% | [25.0% ; 35.0%] | Beta(μ=0.30, σ=0.03) => [25.0% ; 35.3%] |  |
|  | Hearing loss | 33.0% | [28.0% ; 38.0%] | Beta(μ=0.33, σ=0.03) => [28.0% ; 38.2%] |  |
|  | Significant long term neurologic disability | 100.0% | [80.0% ; 100.0%] | Beta(μ=1.00, σ=0.03) => [94.1% ; 100.0%] |  |
| Baseline utility | Aged <25 | 0.92 | [0.89 ; 0.94] | Beta(μ=0.92, σ=0.01) => [0.89 ; 0.94] | Jiang et al. [14]  Range: 95% CI |
|  | Overall population (scenario with herd effect) | 0.851 | [0.839 ; 0.863] | Beta(μ=0.851, σ=0.006) => [0.839 ; 0.863] |  |
| IMD-related disutility | Meningitis | 0.40 | [0.12 ; 0.48] | Beta(μ=0.40, σ=0.16) => [0.12 ; 0.72] | Lecoq et al. [15]  Range: low value equals to assumption in Ortega-Sanchez [2], high value equals to base case +20% |
|  | Septicemia | 0.51 | [0.12 ; 0.61] | Beta(μ=0.51, σ=0.21) => [0.12 ; 0.89] |  |
|  | Other meningococcal infection | 0.40 | [0.12 ; 0.48] | Beta(μ=0.40, σ=0.16) => [0.12 ; 0.72] |  |
| Duration of acute disutility | | 1 year (365.25 days) | [6 months ; 18 months] | Gamma(μ=365.25, σ=109.91) => [182.62 days ; 610.11days] | Ortega-Sanchez [2]  Range: assumption |
| Disutility for IMD cases without sequalae after acute phase | Disutility | 0.03 | [0.02 ; 0.04] | Beta(μ=0.03, σ=0.003) => [0.02 ; 0.04] | Koomen et al. [16] and Schmand et al. [17]  Range: base case ±20% |
|  | Duration | 9 years | [3 years ; 12 years] | Gamma(μ=3,287.25, σ=1,443.14) => [1,095.75 days ; 6,661.10 days] | Koomen et al. [16] and Schmand et al. [17]  Range: assumption |
| Disutility with IMD sequalae | Skin scarring | 0.05 | [0.00 ; 0.18] | Beta(μ=0.05, σ=0.05) => [0.00 ; 0.18] | Ortega-Sanchez [1] |
|  | Single amputation | 0.28 | [0.18 ; 0.63] | Beta(μ=0.28, σ=0.16) => [0.04 ; 0.63] |  |
|  | Multiple amputation | 0.36 | [0.27 ; 0.63] | Beta(μ=0.36, σ=0.13) => [0.13 ; 0.63] |  |
|  | Hearing loss | 0.26 | [0.17 ; 0.33] | Beta(μ=0.26, σ=0.05) => [0.17 ; 0.36] |  |
|  | Significant long term neurologic disability | 0.86 | [0.56 ; 0.92] | Beta(μ=0.86, σ=0.12) => [0.56 ; 1.00] |  |

^a^ The same efficacy against the respective serogroups was assumed for MenABCWY in a scenario analysis

^b^ Lifetime costs

Abbreviations: CDC, Centers for Disease Control and Prevention; DSA, deterministic sensitivity analysis; IMD, invasive meningococcal disease; IRR: incidence rates ratio; PSA, probabilistic sensitivity analysis

# Supplementary Figures

Figure S1: Main analysis - Tornado charts for incremental costs


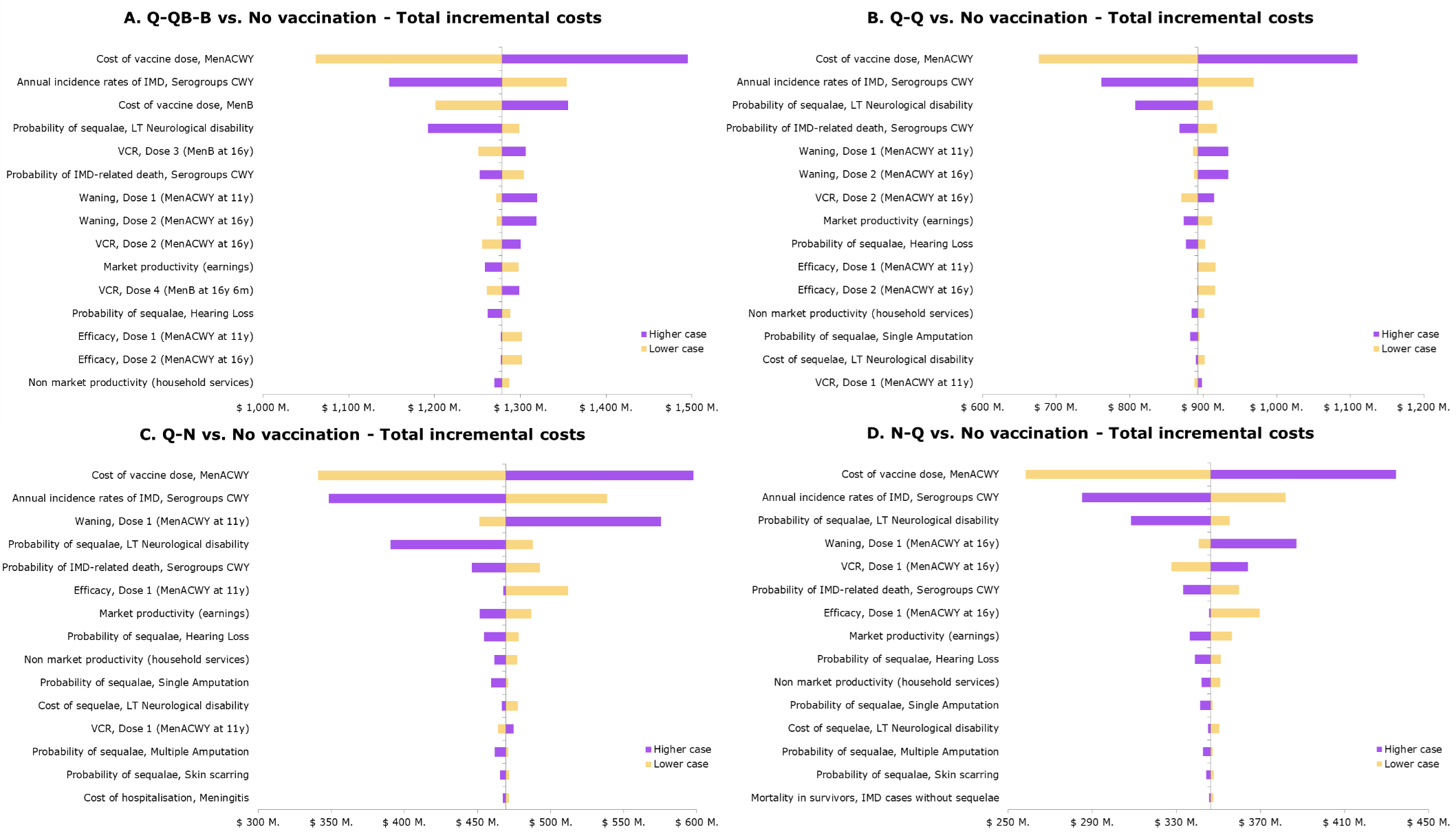


Abbreviations: IMD, invasive meningococcal disease; LT, long-term; VCR, vaccination coverage rate.

Figure S2: Main analysis - Tornado charts for incremental QALYs


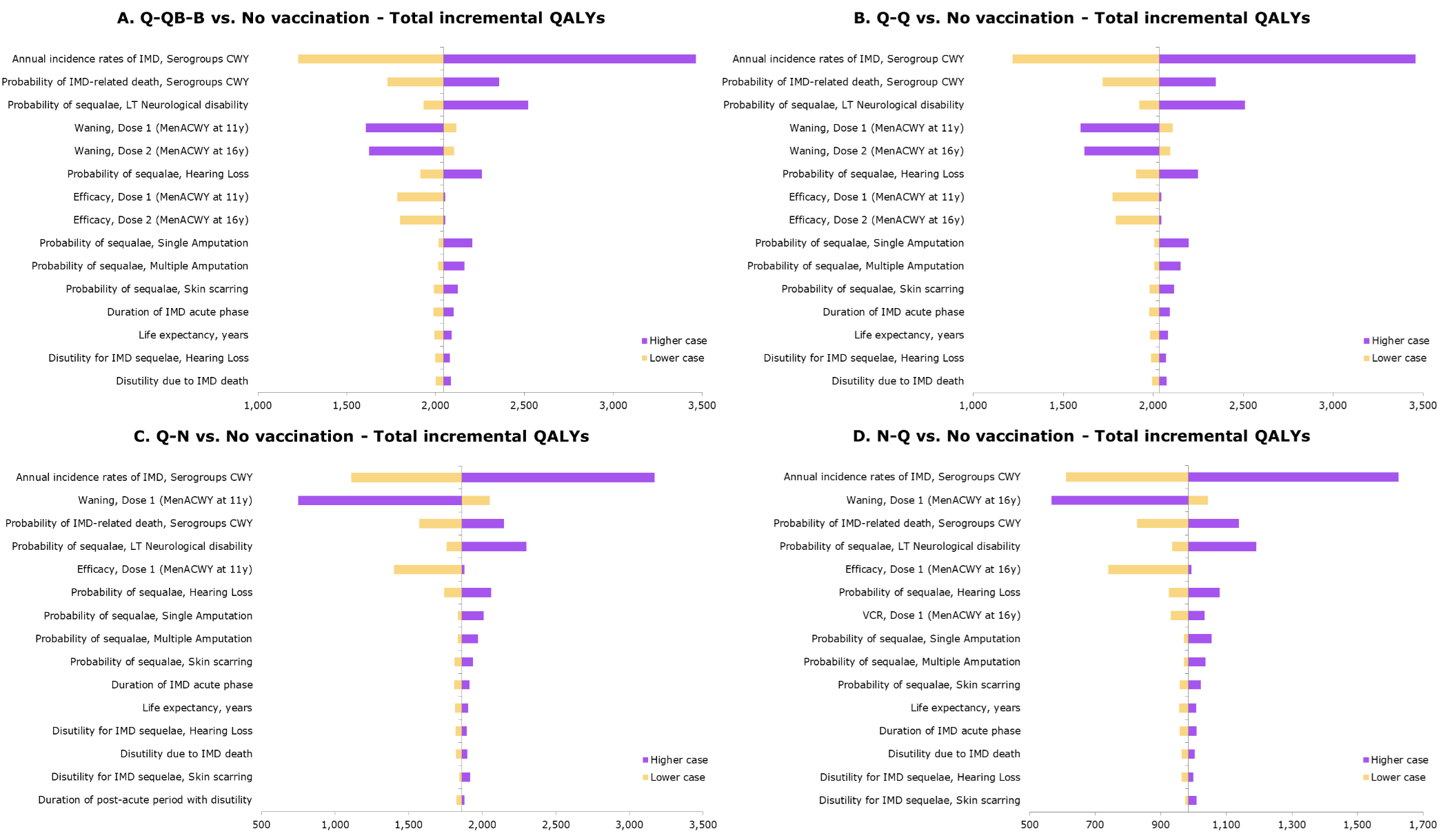


Abbreviations: IMD, invasive meningococcal disease; LT, long-term; QALY, quality-adjusted life year; VCR, vaccination coverage rate.

Figure S3: Scenario analysis - Tornado charts for ICER


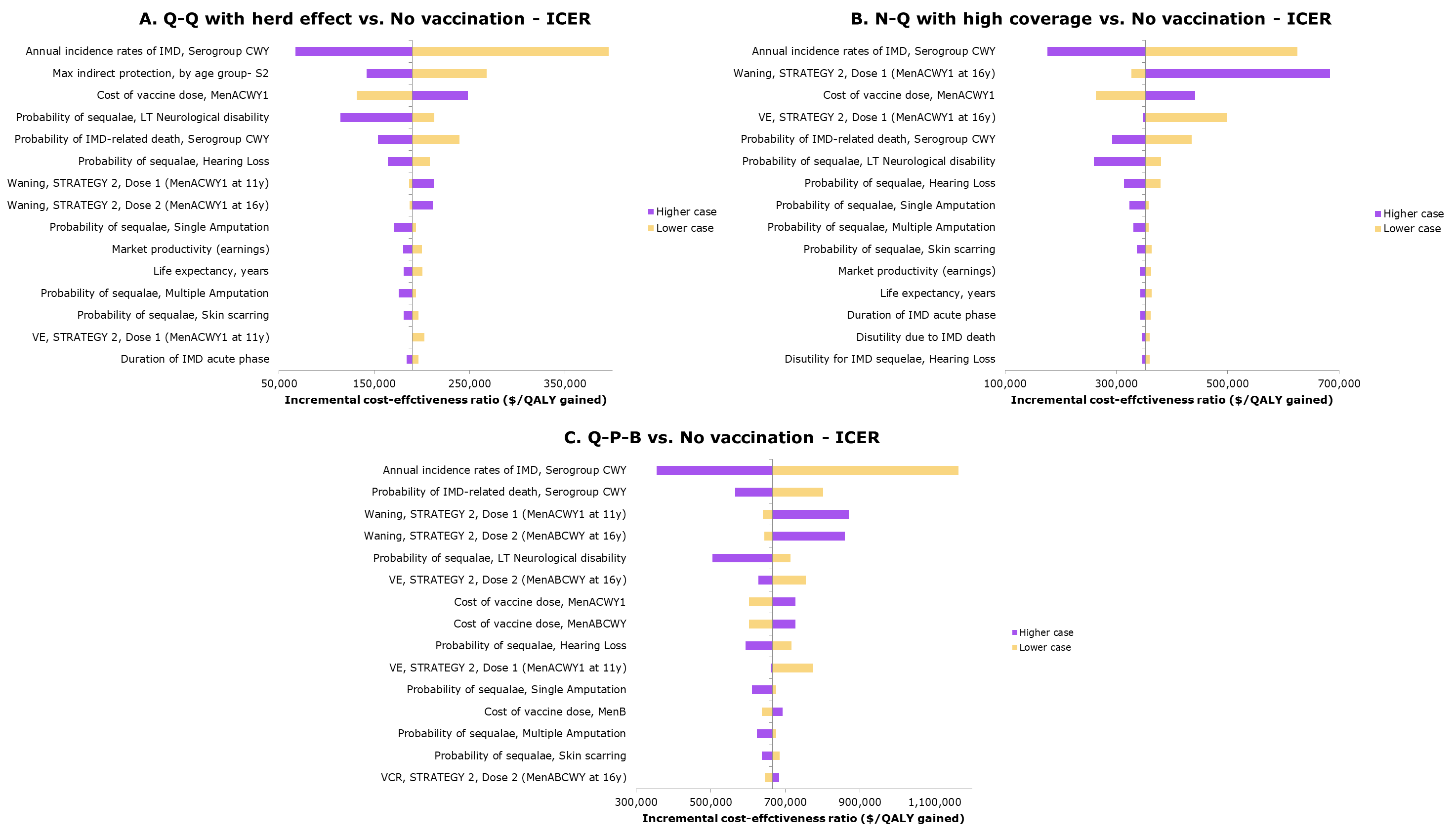


Abbreviations: ICER, incremental cost-effectiveness ratio; IMD, invasive meningococcal disease; LT, long-term; VCR, vaccination coverage rate.

Figure S4: Scenario analysis - Tornado charts for incremental costs


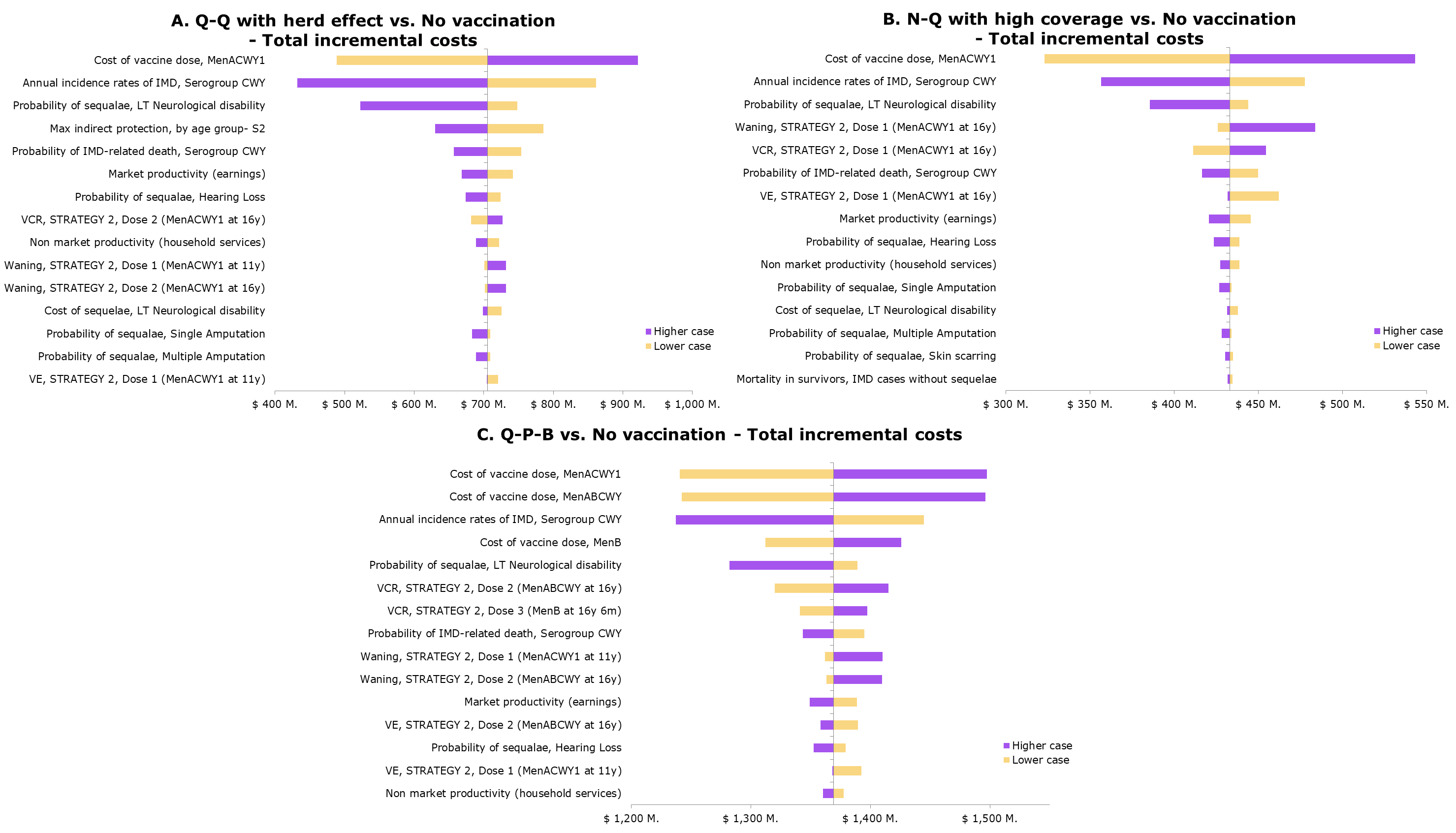


Abbreviations: IMD, invasive meningococcal disease; LT, long-term; VCR, vaccination coverage rate; VE, vaccine efficacy.

Figure S5: Scenario analysis - Tornado charts for incremental QALYs


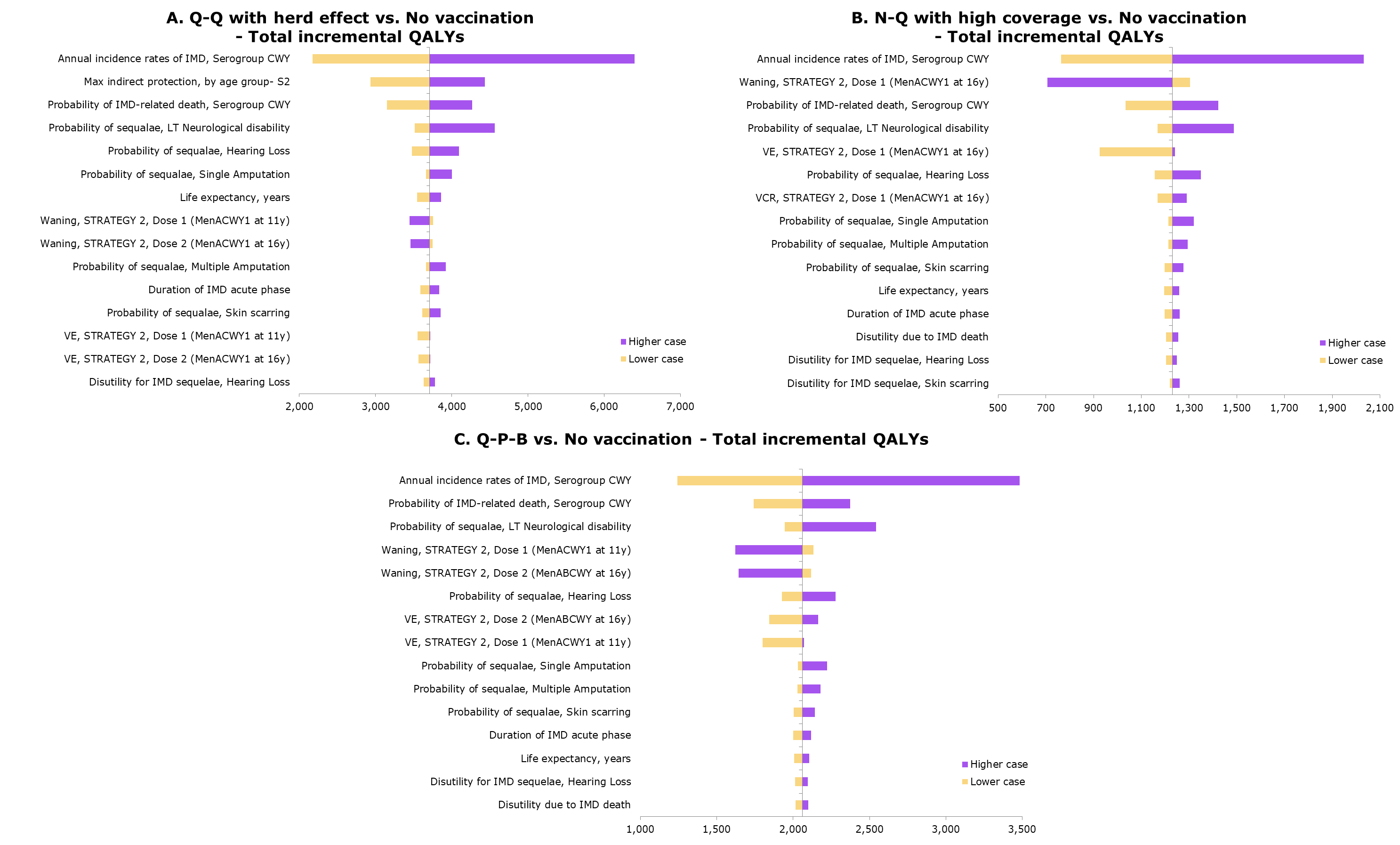


Abbreviations: IMD, invasive meningococcal disease; LT, long-term; QALY, quality-adjusted life year; VE, vaccine efficacy.

Figure S6: PSA of scenarios - Incremental cost-effectiveness planes and cost-effectiveness acceptability curves


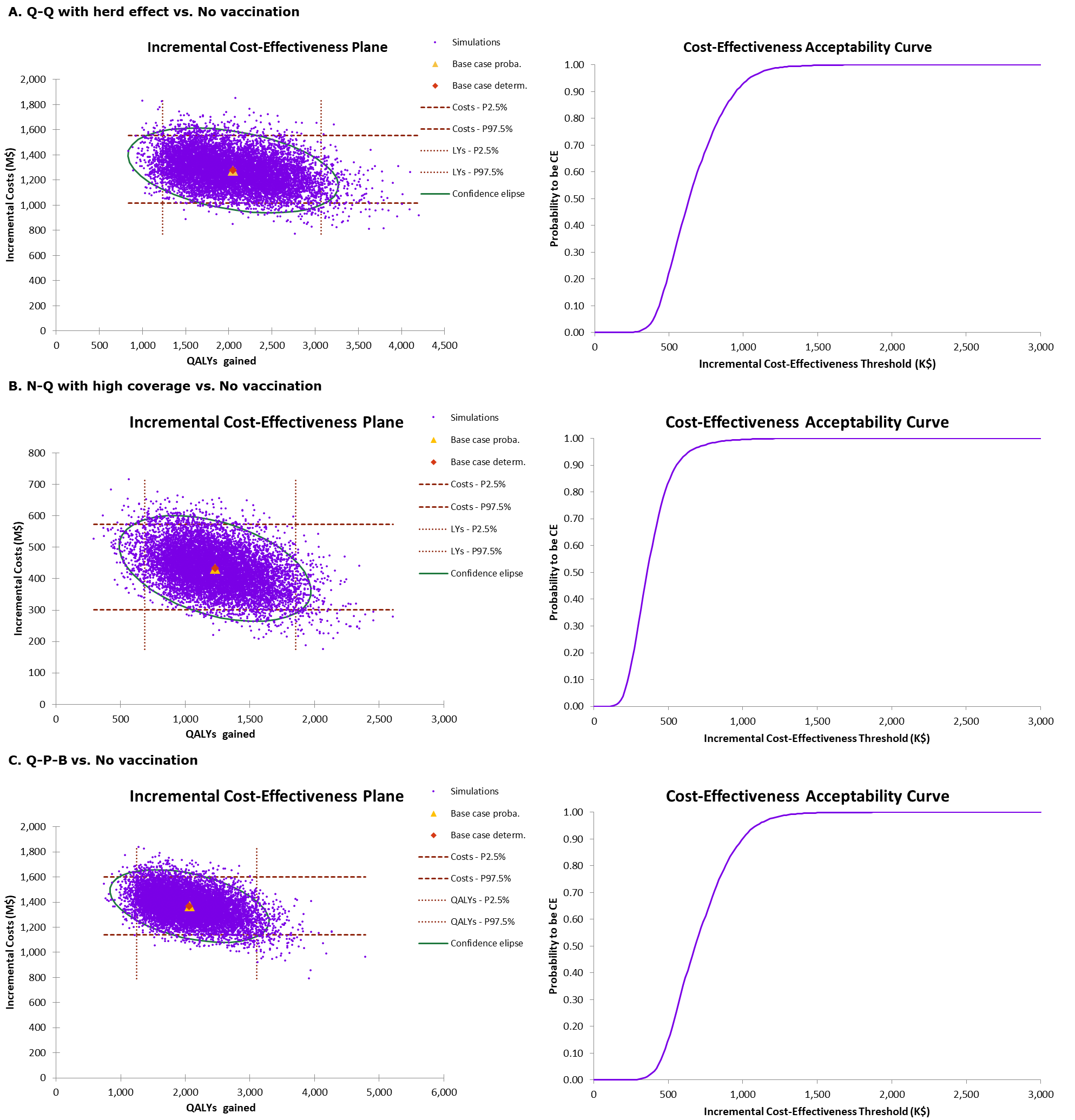


Abbreviations: k, thousand; LY, life-years; M, million; QALY, quality-adjusted life year.

# Supplementary References

1. **The Cost-effectiveness of a Potential Pentavalent Meningococcal Conjugate Vaccine (Men ABCWY) versus the Current Men ACWY and Men B vaccines for US Adolescents** [<https://stacks.cdc.gov/view/cdc/130162>]

2. **Economics of Potential Pentavalent Meningococcal Conjugate Vaccine (MenABCWY) versus the Current MenACWYand MenBvaccines for US Adolescents** [<https://stacks.cdc.gov/view/cdc/134697>]

3. **Population and Housing Unit Estimates Datasets** [<https://www.census.gov/programs-surveys/popest/data/data-sets.2000.List_1725564412.html>]

4. **American Community Survey** [<https://data.census.gov/table/ACSDP5Y2022.DP05?q=United%20States&g=010XX00US>]

5. Arias E, Xu J, Kochanek K: **United States Life Tables, 2021**. *Natl Vital Stat Rep* 2023, **72**(12):1-64.

6. Chang L-J, Hedrick J, Christensen S, Pan J, Jordanov E, Dhingra MS: **A Phase II, randomized, immunogenicity and safety study of a quadrivalent meningococcal conjugate vaccine, MenACYW-TT, in healthy adolescents in the United States**. *Vaccine* 2020, **38**(19):3560-3569.

7. Dhingra MS, Peterson J, Hedrick J, Pan J, Neveu D, Jordanov E: **Immunogenicity, safety and inter-lot consistency of a meningococcal conjugate vaccine (MenACYW-TT) in adolescents and adults: A Phase III randomized study**. *Vaccine* 2020, **38**(33):5194-5201.

8. Zambrano B, Peterson J, Deseda C, Julien K, Spiegel CA, Seyler C, Simon M, Hoki R, Anderson M, Brabec B *et al*: **Quadrivalent meningococcal tetanus toxoid-conjugate booster vaccination in adolescents and adults: phase III randomized study**. *Pediatric Research* 2023, **94**(3):1035-1043.

9. Shepard CW, Ortega-Sanchez IR, Scott RD, 2nd, Rosenstein NE: **Cost-effectiveness of conjugate meningococcal vaccination strategies in the United States**. *Pediatrics* 2005, **115**(5):1220-1232.

10. **Meningococcal Disease. Surveillance** [<https://www.cdc.gov/meningococcal/php/surveillance/?CDC_AAref_Val=https://www.cdc.gov/meningococcal/surveillance/index.html>]

11. Shen J, Bouée S, Aris E, Emery C, Beck EC: **Long-Term Mortality and State Financial Support in Invasive Meningococcal Disease—Real-World Data Analysis Using the French National Claims Database (SNIIRAM)**. *Infectious Diseases and Therapy* 2022, **11**(1):249-262.

12. Sanders GD, Neumann PJ, Basu A, Brock DW, Feeny D, Krahn M, Kuntz KM, Meltzer DO, Owens DK, Prosser LA *et al*: **Recommendations for Conduct, Methodological Practices, and Reporting of Cost-effectiveness Analyses: Second Panel on Cost-Effectiveness in Health and Medicine**. *JAMA* 2016, **316**(10):1093-1103.

13. **ICER’s Reference Case for Economic Evaluations: Principles and Rationale. Current as of January 31, 2020** [<https://icer.org/wp-content/uploads/2020/10/ICER_Reference_Case_013120.pdf>]

14. Jiang R, Janssen MFB, Pickard AS: **US population norms for the EQ-5D-5L and comparison of norms from face-to-face and online samples**. *Qual Life Res* 2021, **30**(3):803-816.

15. Lecocq H, Parent du Châtelet I, Taha MK, Lévy-Bruhl D, Dervaux B: **Epidemiological impact and cost-effectiveness of introducing vaccination against serogroup B meningococcal disease in France**. *Vaccine* 2016, **34**(19):2240-2250.

16. Koomen I, Raat H, Jennekens-Schinkel A, Grobbee DE, Roord JJ, van Furth M: **Academic and behavioral limitations and health-related quality of life in school-age survivors of bacterial meningitis**. *Qual Life Res* 2005, **14**(6):1563-1572.

17. Schmand B, de Bruin E, de Gans J, van de Beek D: **Cognitive functioning and quality of life nine years after bacterial meningitis**. *J Infect* 2010, **61**(4):330-334.

18. Ortega-Sanchez IR, Meltzer MI, Shepard C, Zell E, Messonnier ML, Bilukha O, Zhang X, Stephens DS, Messonnier NE: **Economics of an adolescent meningococcal conjugate vaccination catch-up campaign in the United States**. *Clin Infect Dis* 2008, **46**(1):1-13.

19. Davis KL, Misurski D, Miller JM, Bell TJ, Bapat B: **Cost of acute hospitalization and post-discharge follow-up care for meningococcal disease in the US**. *Hum Vaccin* 2011, **7**(1):96-101.

20. Grosse SD, Krueger KV, Pike J: **Estimated annual and lifetime labor productivity in the United States, 2016: implications for economic evaluations**. *J Med Econ* 2019, **22**(6):501-508.

21. **CDC Vaccine Price List. Updated April 2, 2024** [<https://archive.cdc.gov/#/details?url=https://www.cdc.gov/vaccines/programs/vfc/awardees/vaccine-management/price-list/2024/2024-04-01.html>]

22. Pingali C, Yankey D, Elam-Evans LD, Markowitz LE, Valier MR, Fredua B, Crowe SJ, DeSisto CL, Stokley S, Singleton JA: **Vaccination Coverage Among Adolescents Aged 13-17 Years - National Immunization Survey-Teen, United States, 2022**. *MMWR Morb Mortal Wkly Rep* 2023, **72**(34):912-919.

23. Ramsay ME, Andrews NJ, Trotter CL, Kaczmarski EB, Miller E: **Herd immunity from meningococcal serogroup C conjugate vaccination in England: database analysis**. *Bmj* 2003, **326**(7385):365-366.
